# A surrogate barrier model for high-throughput blood-brain barrier permeability prediction: integrating LLC-PK1-MOCK/MDR1 Cells and lysosomal trapping correction

**DOI:** 10.1080/10717544.2025.2585612

**Published:** 2025-11-26

**Authors:** Juanwen Hu, Xue Jiang, Cong Li, Qiannan Zhang, Xia Wu, Wenpeng Zhang, Xiaomei Zhuang

**Affiliations:** a State Key Laboratory of Toxicology and Medical Countermeasures, Beijing Institute of Pharmacology and Toxicology, Beijing, People's Republic of China

**Keywords:** LLC-PK1-MDR1 cells, blood-brain barrier, CNS drug, surrogate barrier model

## Abstract

To mitigate risks in central nervous system (CNS) drug development, we established a high-throughput in vitro blood-brain barrier (BBB) model using LLC-PK1-MOCK and LLC-PK1-MDR1 cells in a Transwell system, aiming to replicate in vivo brain distribution and elucidate permeability mechanisms. Model integrity was assessed via transepithelial electrical resistance (TEER) and efflux functionality using control drugs (atenolol, digoxin). Bidirectional transport studies of 41 compounds quantified permeability (P_app_), efflux ratios (ER), and recoveries, while in vivo brain distribution parameters (K_*p*,uu,brain_) were derived from literature and rat studies. The model demonstrated critical BBB features: tight junction integrity (TEER > 70 Ω·cm^2^), *P*-gp efflux activity (digoxin ER = 5.10 ~ 17.12), and discrimination of passive diffusion (63.41% of drugs) from transporter-mediated mechanisms (19.5% *P*-gp substrates). A training set of 20 randomly selected drugs revealed a robust correlation between MDR1-derived P_app(A-B)_ and K_*p*,uu,brain_ (R = 0.8886), with the remaining 21 compounds validating predictive accuracy (≤2-fold error). Four alkaloids exhibiting low recovery (<80%) due to lysosomal trapping were corrected using Bafilomycin A1, aligning their permeability with in vivo outcomes. These results position the LLC-PK1-MOCK/MDR1 model as a reliable surrogate tool for early CNS drug screening, enabling rapid prioritization of candidates based on BBB penetration potential. Its integration into preclinical workflows promises to accelerate the development of therapeutics for neurological disorders.

## SIGNIFICANCE STATEMENT

The development of physiologically relevant in vitro BBB models is pivotal to overcoming the high attrition rates in CNS drug discovery. Our study establishes a robust, high-throughput surrogate barrier model using LLC-PK1-MOCK/MDR1 cells in a Transwell system. This model recapitulates critical BBB features, including increased paracellular tightness and *P*-gp transporter functionality. It also addresses longstanding limitations of intracellular drug accumulation by lysosomal trapping. By validating the model with 41 structurally diverse compounds and correlating in vitro permeability (P_app_) to in vivo brain distribution (K_*p*,uu,brain_), we demonstrate its predictive accuracy and utility in distinguishing passive diffusion, transporter-mediated efflux, and lysosomal sequestration mechanisms. This cost and time -efficient platform streamlines early-stage CNS drug screening, enabling rapid identification of brain-penetrant candidates and reducing reliance on resource-intensive in vivo studies.

## Graphical abstract

**Figure:**
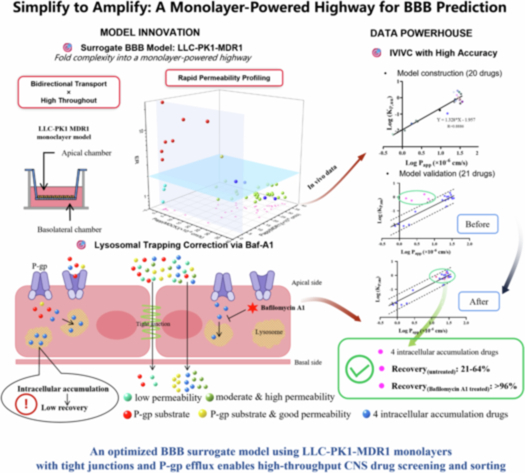

## Introduction

The Blood-Brain Barrier (BBB) is one of the significant obstacles that contribute to the lower success rate of Central Nervous System (CNS) drug development compared to other medications (Cash and Theus 2020; Gil-Martins et al. 2020; Ding et al. 2021). To reduce the risk in the research and development of CNS drugs, establishing a stable, accurate, and high-throughput BBB model for compound screening has consistently been a hot spot and a challenging task in the industry (Kempuraj 2024; Mancuso et al. 2024). Despite studies on the establishment and application of BBB models dates back to the 1970s, and the number of novel BBB models and related literature has surged with the advancement of new technologies over the last two decades, there is still a requirement for robust in vitro models (Shamul et al. 2025). For in vitro models, the criteria for their evaluation must consider the research aims and application scenarios. During the early stage of compound optimization for druggability, it is essential to balance the throughput, accuracy, and cost. Currently, apart from Caco−2 monolayer cells, which has been established as the gold standard in the pharmaceutical industry for evaluating intestinal absorption, there has no widely accepted in vitro BBB model for evaluating transport across BBB (Yan et al. 2021). The quest for the development of dependable models to assess BBB permeability continues to offer both promising prospects and formidable challenges.

The current in vitro BBB models primarily rely on the Transwell system, which is divided into two major categories: non-cellular and cellular models (Terasaki and Tsuji 1994; Han and Jiang 2021; Kawakita 2022). Non-cellular models, such as PAMPA, are characterized by low cost, simplicity, and high throughput. However, their major drawback lies in their limited ability to assess membrane permeability related to passive diffusion mechanisms alone, failing to incorporate the actions of transport proteins expressed in the BBB (Aydogan Avşar and Akkuş 2024). Consequently, these models hampered to realistically simulate the permeability of the BBB in vivo. In contrast, cell-based in vitro BBB models exhibit diversity in their cellular origins, encompassing primary animal or human brain microvascular endothelial cells, immortalized cerebral vascular endothelial cells, and epithelial cell substitutes (Ferro et al. 2020; Wu et al. 2023). Given the scarcity of primary cells, immortalized brain endothelial cells are predominantly utilized in the construction of current in vitro BBB models (Guarino et al. 2023). A prevailing trend in the field of BBB model construction involves the application of cutting-edge technologies such as molecular biology and materials science to fully recreate the structural and physiological state of the authentic BBB. This has led to the establishment of 2D-BBB models involving co-cultures of brain microvascular endothelial cells with astrocytes, microglia and pericytes, as well as 3D microfluidic BBB models (Cui and Cho 2022; Bi et al. 2023). Furthermore, stem cell-inducible techniques have been employed to create co-culture models incorporating humanized vascular endothelial cells, astrocytes, and more (Wang 2024; Mancuso et al. 2024). Published epithelial cell surrogate models for BBB research primarily encompass Caco−2 cells, ECV304 cells, MDCK-MDR1 single-transfected cells, and MDCK-MDR1/BCRP double-transfected cells (Hellinger et al. 2012; Dithmer et al. 2024). Epithelial cell models offer several advantages over cerebral vascular endothelial cells, including simple culture conditions, rapid growth duration, and consistent reproducibility (Balzer 2022). Among these, MDCK cells are particularly noted for their robust intercellular junctions, establishing them as a widely accepted tool for the development of efflux protein-transfected models (Ozgür 2021; Fang 2023; Colclough 2024). Despite these benefits, discrepancies in reported data across various laboratories still exist (Ponmozhi et al. 2024). Furthermore, there is a current absence of comprehensive reports detailing the optimization of model conditions, the specific characteristics of these models, and their correlation with in vivo brain bio distribution (Gao 2016; Alluri et al. 2024; Lim et al. 2024). Given the practical requirements for early-stage compound screening in BBB permeability assessment, it is necessary to develop a simplified BBB model (Sánchez-Dengra et al. 2023). Such a model should be cost-effective, capable of high-throughput screening, and possess a good relevance to in vivo conditions, leveraging epithelial cells as surrogate models. This approach could bridge the gap in our current understanding and facilitate more accurate predictions of drug permeability across the BBB.

LLC-PK1 cells, which are immortalized porcine kidney epithelial cells originated from 1970‘s, exhibited good cell tightness due to the expression of tight junction proteins. After transfection with the MDR1 gene, these cells stably and highly express the *P*-gp, making them useful for investing the interactions between drugs and *P*-gp (Sóskuti 2024). Considering that LLC-PK1-MDR1 cells can form a highly dense monolayer that prevents the passage of *P*-gp substrate through the cells (Sato et al. 2020). We characterized the model's key functional properties, with a focus on barrier integrity (paracellular tightness) and *P*-gp transporter activity, using highly sensitive detection techniques and in vitro-in vivo comparison (Schreiner et al. 2022). After validation of the robustness via 41 various types of drugs, a complete and reproducible technical process and key parameter judgment criteria for regular laboratories to rapidly and accurately estimate the BBB permeability properties of compounds were achieved. Thereby, efficiently facilitate the capability and proficiency in druggability assessment.

## Materials and methods

### Agents and compounds

1640 basic culture medium (C11875500BT), fetal bovine serum (FBS, A5669701), and penicillin-streptomycin mixture (15140122) were purchased from Gibco (US). Hank’s balanced salt solution (HBSS, H1025), lidocaine (SL8860), and salbutamol (SS9980) were purchased from Solarbio (China). Lucifer yellow CH dipotassium salt (LY, 9456305) was purchased from J&K Scientific (US). Tariquidar (S80527) was purchased from Shanghai Yuanye Biotechnology Co., Ltd. (China). Anti rabbit IgG (whole molecule)-FITC goat antibody (F0382), dimethyl sulfoxide (DMSO, D2650) purchased from Sigma-Aldrich (US). Claudin 7(349100), Occludin (711500), ZO−1 (677300), and *P*-gp rabbit clone antibodies (MA5−13854) were purchased from Invitrogen (US). Propranolol (S4076), digoxin (S4290), atenolol (S4817), metoprolol (S1856), dexmedetomidine (S2090), methotrexate (S1210), phenacetin (S2577), bupropion (S2542), verapamil (S4202), and omeprazole (S1389) were purchased from Selleckchem (US). Caffeine (9201) was purchased from the National Narcotics Laboratory (China). Chlorzoxazone (C4397), quinidine (Q3625), and dextromethorphan (D9684) were purchased from Sigma (US). Antipyrine (B1886), lamotrigine (B2249), amitriptyline hydrochloride (B2231), etoposide (A1971), and cimetidine (B1557) were purchased from APExBIO (US). Doxorubicin (HY-15142A), tacrine (HY-B2244), and bafilomycin A1 (A8627-5A) were purchased from MedChemExpress (US). Tramadol hydrochloride (C10125000) was purchased from Dr. Ehrenstorfer GmbH (Germany). Tolbutamide (46968) was purchased from Fluka (Germany). Sunitinib (S126061), vinblastine (V127032), and paroxetine hydrochloride (P304169) were purchased from Shanghai Aladdin Biochemical Technology Co., Ltd (China). Indomethacin (GC17556) was purchased from Glpbio (US). Diphenhydramine hydrochloride (D832909) and melatonin (M874109) were purchased from Shanghai Macklin Biochemical Technology Co., Ltd (China). Warfarin (B25391) was purchased from the China Institute of Pharmaceutical Biometry (China). Cannabinoid, 1655, 705, 1111, tetrandrine, modafinil, nimodipine, atipemazole hydrochloride, and midazolam were all synthesized in-house by Beijing Institute of Pharmacology and Toxicology (China).

### Cells lines

LLC-PK1 cell with stable expressing human MDR1 and mock-transfected with empty vector (LLC-PK1 MOCK) were gifted by the lab of Professor Qingcheng Mao (University of Washington, Seattle, WA, USA). The overexpression and functional activity of *P*-glycoprotein (*P*-gp) in the LLC-PK1-MDR1 cells were rigorously validated through multiple methods. Significantly increased MDR1 mRNA expression levels were confirmed by quantitative real-time PCR, and increased *P*-gp protein expression on the apical membrane of the LLC-PK1-MDR1 monolayers was confirmed by immunofluorescence staining, as detailed in Section 2.5. The functional activity of *P*-gp was confirmed by demonstrating polarized (basolateral-to-apical), saturable efflux of known *P*-gp substrate drugs (digoxin). The efflux ratio (P_app,B-A_/P_app,A-B_) for these substrates was significantly higher in LLC-PK1-MDR1 cells compared to the LLC-PK1 MOCK cells.

### Animals

135 adult male SD rats, 200–230g, were purchased from Beijing Viton Lihua Laboratory Animal Science and Technology Co. Animals were housed in a clean facility with a 12-h light/dark cycle and fed food and water ad libitum at 22 °C and 50% relative humidity. At the end of the study, the rats were euthanized by exposure to an increasing concentration of CO_2_. No abnormal clinical symptoms were observed during any of the experiments in the SD rats. All experiments followed the guidelines of the International Association for Assessment and Accreditation of Laboratory Animal Care (AAALAC) and were conducted in the Beijing Institute of Pharmacology and Toxicology following the ethical approval of the Beijing Institute of Pharmacology and Toxicology's Animal Care and Use Committee (IACUC-DWZX−2023-P665).

### Cell culture and transport protocol optimization

LLC-PK1 MOCK and LLC-PK1 MDR1 cells were cultured at 37 °C in a 5% CO_2_ atmosphere using 1640 medium supplemented with 10% fetal bovine serum (FBS) and 100 U/mL penicillin-streptomycin solution. Cells were passaged at 80−90% confluence (every 2−3 days) using trypsin-EDTA solution. For bidirectional transport studies, LLC-PK1 MOCK cells were seeded in the inserts at a density of 2 × 10^4^ cell/well in Millicell 24-well cell culture plates with a 6.5 mm insert (0.4 µm polycarbonate membrane) from Corning (3413, Kennebunk, ME, United States). The culture medium was replaced every other day, including 200 µL culture medium in the apical and 850 µL cell culture medium in the basolateral side. LLC-PK1 MOCK cells were used at passages of 3−30. LLC-PK1-MDR1 cells were cultured and passaged in the same manner as LLC-PK1 cells except at the density of 1 × 10^4^ cells per well. LLC-PK1-MDR1 cells were used at passages of 3−25. For LLC-PK1 MOCK cells and LLC-PK1-MDR1 cells, the experiments were performed on the 4^th^ and 7^th^ day after seeding.

### Validation of MDR1 expression and Barrier properties

**Detection of ZO−1, Claudin−7, Occludin, and *P*-gp protein expression using immunostaining.** ZO−1, Claudin−7, Occludin, and *P*-gp protein expression on the LLC-PK1 MOCK/MDR1 cell model were examined by immunofluorescence labeling procedures. We optimized the sampling time for each cell line, taking into account their unique growth and differentiation kinetics, to ensure that images were captured when tight junction proteins were fully expressed and the barrier functionally mature. Specifically, immunofluorescence staining was conducted on day 3 for LLC-PK1 MOCK cells and on day 6 for LLC-PK1-MDR1 cells.The samples were fixed using -20 °C methanol for 20 min, then washed with DPBS. After that, 10% goat serum (diluted in DPBS) was added to each well as a blocking buffer and incubated with shaking at room temperature for 2 h. Claudin−7 rabbit polyclonal antibody, Occludin rabbit polyclonal antibody, ZO−1 rabbit polyclonal antibody, *P*-gp rabbit polyclonal antibody were used as primary antibodies in 1:500, and then added to the samples, which were placed into the incubation at 4 °C overnight. Following a brief rinse in DPBS, the samples were incubated using the secondary antibody, a FITC-conjugated goat, anti-rabbit IgG in a dilution of 1:500 for 2 h at room temperature in the dark. Wash off with PBS at the end of the incubation. Cell nuclei were stained using 10 µg/mL DAPI dye for 10 min, washed three times with PBS at the end of the incubation, and then blocked using an anti-fluorescence quencher.

**Monitoring of TEER value and LY permeability**. After the LLC-PK1 MOCK/MDR1 cells attach to the Transwell insert, TEER (Transepithelial Electrical Resistance) values were continuously recorded using a cell resistance meter (Beijing JINGONGHONGTAI Technology Co., Ltd). These TEER values represent the mean from six individual inserts (*n* = 6) measured per group. The TEER measurement and transport experiment were conducted at 37 °C, using a heating plate to maintain the required temperature. The transmembrane resistance was calculated using the formula TEER = (R_total_-R_blank_) × A_area_, where R_blank_ represents the TEER of the blank chamber.

The permeability of the Lucifer Yellow CH Dipotassium Salt (LY, 100 μM) across the monolayers was measured in apical-to-basolateral direction to monitor the integrity of the cell monolayer. The fluorescence was measured at 428 nm excitation wavelength and 540 nm emission wavelength using a microplate reader to calculate P_app_ values (Qi 2016).

**Detection of MDR1 gene expression using qPCR**. Total RNA from confluent LLC-PK1 MOCK and LLC-PK1-MDR1 cells monolayers was extracted using TaKaRa MiniBEST Kit (9767, TaKaRa, Japan) according to the manufacturer’s instructions. Complementary DNA (cDNA) was synthesized from the extracted RNA by reverse transcription using the PrimeScript PLUS RTase kit (F0202, LABLEAD, China). The gene expression of MDR1 was then evaluated by quantitative real-time PCR (qPCR) using TaKaRa Ex Taq HS Mix on the resulting cDNA. The primer sequences were as follows: 5’-CATCAACTTTCCGGGGGTGA−3’ (forward) and 5’-CACTGGTTGGTCGTCAGGAA−3’ (reverse) for MDR1; 5’-ATCAACGGGAAGTCCATCTCC−3’ (forward) and 5’-ATGGTTCACGCCCATCACAA−3’ (reverse) for probe GAPDH. Thermocycling was carried out with 5 minutes at 42 °C/10 seconds at 95 °C for reverse transcription, followed by 40 cycles of 5 seconds at 95 °C and 34 seconds at 60 °C. Each sample was analyzed in triplicate.

**Monitoring permeability of positive control drugs**. DMSO mixed stocks (10 mM) of positive control drugs (atenolol and metoprolol) were diluted in HBSS (containing 0.1% BSA and 10 mM Hepes) pH 7.4 to achieve a working solution of 2 µM. The Transwell media inserts were washed by 37 °C pre-warmed HBSS (containing 0.1% BSA and 10 mM Hepes) buffer and then preincubated at 37 °C in 5% CO_2_ for 30 minutes. The permeability of test compounds at LLC-PK1 MOCK and LLC-PK1-MDR1 cells monolayers over 2 h were measured in both directions and the receiver wells contained HBSS (containing 1% BSA and 10 mM Hepes) buffer. Samples were collected from the donor well at the start of the incubation (50 µL) as C_0_, and from the donor and receiver wells at the end of the incubation (50 µL). All samples were stored at - 40 °C for LC-MS/MS analysis.

To assess the function of *P*-gp in the LLC-PK1-MDR1 cells line, transport assays of digoxin were run at 2 µM in both directions in the presence and absence of the *P*-gp inhibitor tariquidar at 5 µM.

### Barrier bidirectional transport studies of 41 drugs

**Cell Counting Kit−8 (CCK−8) assay**. Cell viability was determined by CCK−8 assay following the protocols of manufacturer. LLC-PK1 MOCK/MDR1 cells were seeded into 96-well plates at a density of 1 × 10^4^ cells per well in 100 µL of culture medium and cultured overnight. All drugs were added into 96-well plates at final concentration of 2 µM. After 2 h incubation, 10 µL of CCK−8 solution (CK05, Beyotime, China) was added into each well. The plates were then incubated for another 2 h in the incubator. The optical density values were recorded at 450 nm using a microplate reader (Thermo Fisher Scientific). Data from five replicates were presented as the percentage of treated cells relative to untreated controls.

**Bidirectional transport experiments**. DMSO stocks (10 mM) of test compounds were diluted in HBSS (containing 0.1% BSA and 10 mM Hepes) pH 7.4 to achieve a working solution of 2 µM (Based on preliminary experimental results, the optimized 2 µM features no cytotoxicity, falls within the linear membrane permeability range of most compounds, and is easy to detect, etc). The transwell experiments were conducted following above-mentioned procedure. A set of positive control drugs (atenolol, metoprolol and digoxin) were carried out with each batch of bidirectional transport experiments to monitor the integrity of the monolayer cell model barrier and the efflux effect of *P*-gp. When the recovery rate of the tested drug is below 80%, the possibility of intracellular accumulation should be considered. The polycarbonate (PC) membrane where cells are grown should be carefully peeled off from the cell culture medium and placed in 100 µL of HBSS buffer solution. Subsequently, the cell monolayers were lysed, and the samples were subjected to ultrasonication using an ultrasonic cell disruptor (KUNSHAN ULTRASONIC INSTRUMENT CO. LTD, KQ-300DE). The process was carried out on ice to prevent heat-induced degradation, applying a power in the range of 300 W for a total duration of 20 minutes. Thereafter, a quantitative analysis of the intracellular drug was conducted, and the drug recovery rate was recalculated based on this analysis.

The apparent permeability coefficient (P_app_, cm/s) was calculated for each transport direction, A-B and B-A, using the following equation:

$$P_{\mathrm{app}}=\frac{\mathrm{dQ}}{\mathrm{dt}}\times \frac{1}{A\times C_{0}}$$

where dQ/dt is the permeation rate (µmol/s), A is the surface area of the insert (0.33 cm^2^, Corning), and C_0_ is the initial donor concentration (µM).

The efflux ratio (ER) is calculated as the ratio of Papp (B-A)/Papp (A-B).

$$\mathrm{Recovery}=\frac{\mathrm{TRcumrf}+\mathrm{Cdf}\times \mathrm{VD}}{\mathrm{Cdi}\times \mathrm{VD}}\times 100\%$$

Where TRcumrf is the cumulative transported mass in the receiver chamber at the end of the incubation (nmol), Cdf is the concentration of the drug in the donor chamber at the end of the incubation (μM), VD is the volume of the donor chamber (mL), and Cdi is the initial concentration of the drug in the donor chamber (μM).

**Experimental assessment of transmembrane transport of four drugs after inhibiting intracellular accumulation.** Based on the results of the bidirectional transport experiments of the above-mentioned test drugs, for the drugs that caused intracellular accumulation, bafilomycin A1 was added to inhibit lysosomal function in the transmembrane transport experiments of the LLC-PK1 MOCK/MDR1 monolayer cell model. The method of the bidirectional transport experiment was the same as before. In addition to adding the test drug and the receiving solution to the upper and lower chambers, 1 µM Bafilomycin A1 was also added.

### Brain distribution assessment of 41 drugs in rats

In vivo unbound brain distribution data (K_*p*,uu,Brain_) for 16 drugs and total brain distribution data (K_*p*,Brain_) for 16 drugs was acquired from literature. For the remaining 9 drugs (dextromethorphan, sunitinib, etoposide, doxorubicin, modafinil, antipyrine, vinblastine, phenacetin, and cimetidine), the brain exposure studies were conducted in healthy SD rats. For each tested compound, fifteen rats were randomly divided into five groups of three rats each. The 135 rats were injected intravenously at 1 mg/kg of the compound (dissolved in saline to make a 0.2 mg/mL solution). The time of execution of the rats by CO_2_ inhalation after administration was optimized according to the in vivo elimination rate to achieve five concentrations of different time points. After the rats were euthanized, cardiac puncture was performed immediately to remove as much whole blood as possible (~7 ml) from the systemic circulation (Quantitatively aliquot 0.2 ml of whole blood for the determination of drug concentration in plasma). Afterwards, the brain tissue was collected immediately and rinsed repeatedly with ice-cold saline 3 times. Then, the surface saline was blotted dry with clean filter paper to remove as much residual drug in the blood vessels as possible. Plasma samples were prepared by centrifugation of the blood samples at 2,500 g for 10 minutes at 4 °C. All brain samples were homogenized by adding 4-fold volume water. All samples were stored at -40 °C before LC-MS/MS analysis. Statistical analysis of absolute drug concentrations in plasma and brain tissue was conducted using GraphPad v8, data displayed as mean ± SD. The brain tissue partition coefficient (K_*p*, brain_) and brain-to-plasma unbound drug partition ratio (K_*p*,uu, brain_) are calculated according to the following formulas, respectively:

$${\text{K}}_{\text{p},\;\text{brain}}\;=\;{\text{AUC}}_{\left(0-\text{∞},\text{brain}\right)}/{\text{AUC}}_{\left(0-\text{∞},\text{plasma}\right)}K_{p,\mathrm{uu},\mathrm{brain}}=K_{p,\mathrm{brain}}\times (f_{u,\mathrm{tissue}}/f_{u,\mathrm{plasma}})$$

### Free fraction of 41 drugs in rat plasma and brain protein

Fraction unbound of test compounds in rat plasma (f_u,plasma_, omeprazole, paroxetine, caffeine, lidocaine, salbutamol, vincristine, sunitinib, cimetidine, phenacetin, dexamethasone, amitriptyline, and S-071031B) and brain homogenate (f_u,brain_, 1111, 705, midazolam, nimodipine, omeprazole, paroxetine, caffeine, 1655, lidocaine, salbutamol, vincristine, doxorubicin, sunitinib, etoposide, cimetidine, phenacetin, modafinil, dexamethasone, antipyrine, and S-071031B) was measured by equilibrium dialysis.

Stocks (10 mM) of test compounds were diluted to 5 μM with plasma or tissue homogenate and dialyzed at 37 °C for 6 hours in a shaking incubator. After incubation aliquots of both plasma or tissue homogenate and buffer from the device were taken and added to equal volumes of blank buffer and plasma or tissue homogenate respectively. Samples were then diluted in acetonitrile containing an internal standard prior to centrifugation and analysis of the supernatants by LC-MS/MS. The f_u, plasma_ was calculated using Equation 1:

(1) $${\text{f}}_{\text{u},\;\text{plasma}}\;={\;\text{Conc}}_{\text{buffer chamber}}/{\text{Conc}}_{\text{sample chamber}}$$

Rat fraction unbound in brain (f_u, brain_) was determined as the ratio of concentration in buffer to that in brain. Undiluted f_u, brain_ was calculated with correction for the dilution factor (D) (Wan et al. 2007):

(2) $$\text{Undiluted}\;{\text{f}}_{\text{u},\;\text{tissue}\;}=\;\frac{1/\text{D}}{((1/{\text{f}}_{\text{u},\;\text{apparent}})-1)+1/\text{D}}$$

### Determination by LC-MS/MS

All samples of tissue homogenate, plasma, cell lysate, and cell culture medium were precipitated with acetonitrile (containing internal standard, propranolol, or tolbutamide, 10 ng/mL). After centrifugation at 4 °C, 18,800 g for 10 minutes, the supernatant was diluted twice with 50% acetonitrile prior to injection on the LC-MS/MS. Each batch of samples is accompanied by a calibration curve during assessment.

### Correlation analysis

To establish and validate the correlation between the measured apparent permeability coefficients of test drugs obtained in LLC-PK1 MOCK/MDR1 monolayer cells and in vivo brain distribution data, initially, 20 test drugs were randomly selected from 36 drugs with recoveries above 80% to serve as the training set. To minimize bias in selecting 20 compounds for establishing the in vivo-in vitro correlation, we used a stratified random method: randomly selecting from compounds with varying permeability, ensuring they cover diverse permeability traits and mechanisms. A linear correlation analysis was conducted between the P_app(A-B)_ and the K_*p*,uu,brain_ values. The remaining test drugs were utilized as a validation set to ascertain the accuracy of the model. In ADMET studies, parameters like BBB permeability often follow nonlinear (e.g. exponential) relationships. Applying linear regression to raw data can underestimate R² due to this nonlinearity. Log transformation linearizes these trends, resulting in higher and more representative R² values. In fact, similar studies (Sato et al. 2020; Colclough 2024) typically use log-transformed data for linear regression.

## Results

### Features of surrogate BBB monolayer model

#### Morphometric analysis of monolayer organization parallels Barrier-associated gene and protein expression dynamics

**Figure 1.**
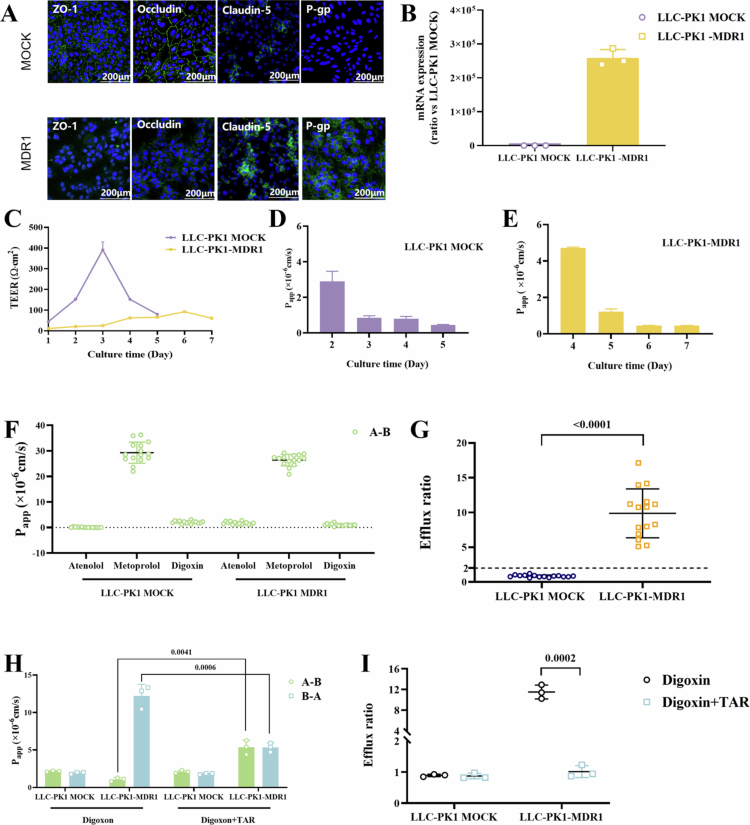
Features of this surrogate BBB monolayer model (A) Tight junction proteins and *P*-gp expression levels of LLC-PK1 MOCK/MDR1 cells (The green fluorescence represents tight junction protein and *P*-glycoprotein, while the blue fluorescence represents the nucleus, located in the cytoplasmic center); (B) Expression levels of MDR1 in LLC-PK1 MOCK/MDR1 cells (mean ± s, *n* = 3); (C) Changes in TEER values of LLC-PK1 MOCK/MDR1 cell model with culture days (mean ± s, *n* = 6); (D) Changes in A-B permeability of LY in LLC-PK1 MOCK cell model over culture time (mean ± s, *n* = 3); (E) Changes in A-B permeability of LY in LLC-PK1-MDR1 cell model over culture time (mean ± s, *n* = 3); (F) Permeability of positive control drugs in LLC-PK1 MOCK/MDR1 cells (mean ± s, *n* = 15); (G) Efflux rate of positive control drugs in LLC-PK1 MOCK/MDR1 cells (mean ± s, *n* = 15); (H) Permeability of digoxin in LLC-PK1 MOCK/MDR1 cell models with and without the *P*-gp inhibitor (TAR) (mean ± s, *n* = 3) (I) Efflux rate of digoxin in LLC-PK1 MOCK/MDR1 cell models with and without the *P*-gp inhibitor (TAR) (mean ± s, *n* = 3).

Observed under an inverted microscope, both LLC-PK1 MOCK cells and LLC-PK1-MDR1 monolayer cells presented a cobblestone-like structure. After immunofluorescence staining of tight junction proteins, observations were carried out using a laser scanning confocal microscope. The results demonstrated that Claudin 7, Occludin, and ZO−1 were highly expressed on LLC-PK1 MOCK cells. Whereas LLC-PK1-MDR1 cells only expressed Occludin and ZO−1, with reduced levels of expression. Moreover, *P*-gp was highly expressed in LLC-PK1-MDR1 cells, while no expression of *P*-gp was detected in LLC-PK1 MOCK cells (Figure 1A). The results of qPCR analysis revealed that MDR1 was highly expressed in LLC-PK1-MDR1 cells, whereas the content of MDR1 in LLC-PK1 MOCK cells was extremely low (Figure 1B).

#### TEER dynamics and their correlation with epithelial monolayer barrier function

The TEER of both cell monolayers was dynamically monitored throughout the culture period using a Millicell ERS volt-ohm meter. As shown in Figure 1C, distinct temporal patterns emerged between the two cell models. The LLC-PK1 MOCK control cells demonstrated transient barrier formation, with TEER values peaking at 400 Ω·cm² on day 3 post-seeding before progressively declining to a stable baseline of 100 Ω·cm². In contrast, LLC-PK1-MDR1 monolayers exhibited delayed barrier development, showing gradual TEER elevation during the first 6 days of culture. This slow maturation culminated in a maximum resistance of 100 Ω·cm² at day 6, followed by moderate decrease in subsequent measurements. The integrity of the monolayer cell barrier was further verified by measuring the P_app_ of the poorly permeable drug LY transported potentially via the paracellular route. According to literature reports, a successful establishment of a dense monolayer barrier can be considered when the P_app_ value of LY is within the range of 0.2 × 10^−6^ to 2 × 10^−6^ cm/s (Gao 2020). The results indicated that the LLC-PK1 MOCK and LLC-PK1-MDR1 cell models met the barrier requirements on the third and sixth days, respectively, and were able to maintain this state for 2−3 days (Figure 1D, E). Repeated validation showed that the decrease in cell resistance values after reaching a peak did not impact the density of the model barrier. To avoid batch-to-batch variability and ensure that the models had reached the required compactness for the transport experiments, it was determined that transport experiments would be conducted on the fourth day for the LLC-PK1 cell model and the seventh day for the LLC-PK1-MDR1 cell model.

In addition to LY, we also selected atenolol (a low permeability drug that travels passively through the paracellular pathway), and metoprolol (a high permeability drug that travels passively through the cell membrane), and digoxin (a specific substrate of *P*-gp), as the cock-tailed positive control drugs for the bidirectional transport experiments (Gao 2020). The detailed results of the bidirectional transport experiments are displayed in Figure 1F and G. The permeability of atenolol was extremely low in both the LLC-PK1 MOCK and LLC-PK1-MDR1 cell models, with the P_app (A-B)_ values being 0.29 × 10^−^⁶ cm/s and 1.73 × 10^−^⁶ cm/s respectively. Meanwhile, the P_app (A-B)_ values of metoprolol in both models exceeded 20 × 10^−^⁶ cm/s, confirming the integrity and barrier efficacy of the constructed monolayer models.

To assess the function of *P*-gp in LLC-PK MOCK/MDR1 cell models, bidirectional transport assays of digoxin were conducted in the absence and presence of *P*-gp inhibitor tariquidar (5 µM) (Figure 1H, I). Digoxin exhibited minimal directional transport (ER = 0.89 ± 0.04) in LLC-PK1 MOCK monolayers, whereas LLC-PK1-MDR1 cells demonstrated robust efflux capacity with an ER of 11.52 ± 1.34 (*p* < 0.001). Pharmacological inhibition with 5 μM tariquidar, a selective *P*-gp antagonist, completely abolished this polarized transport in MDR1-expressing cells - the ER plummeted from 11.52 to 0.99, accompanied by a 3.97-fold increase in apical-to-basolateral (A→B) permeability. Notably, tariquidar treatment in MOCK cells showed no significant alterations in either permeability parameters (2.17 × 10^−^⁶ cm/s vs 2.10 × 10^−^⁶ cm/s, *p* > 0.05) or ER values (0.89 vs 0.87, *p* > 0.05). These results validated that the LLC-PK1-MDR1 model maintains functional *P*-gp-mediated efflux activity through stable and polarized expression of the transporter protein, while the parental MOCK line lacks detectable *P*-gp functionality.

### Barrier permeability and transport mechanism profiling of 41 test drugs

**Transmembrane transport characteristics of positive drugs and 41 test drugs**. Using the validated LLC-PK1 MOCK/MDR1 dual-cell model, we systematically profiled the bidirectional transmembrane permeability of 41 compounds. To ensure data integrity, each experimental batch incorporated three control drugs (atenolol, metoprolol, and digoxin). LC-MS/MS was employed to measure the drug concentrations in the donor chamber and the receiver chamber. The transport experimental results (P_app(A-B)_ and ER) of the positive control drugs in each batch were summarized in Figures 1F, G, which confirmed that all batches (with a CV < 14.0%) meet the predefined acceptance criteria (CV < 15.0%) (Vazvaei-Smith et al. 2024).

**Figure 2.**
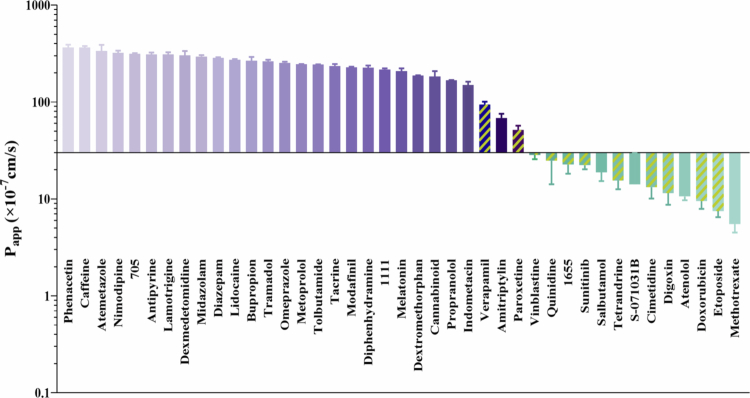
The P_app(A-B)_ value of 41 tested drugs in LLC-PK1-MDR1 cell model. The purple columns represent compounds with high permeability, the green columns represent compounds with low permeability, and the yellow shaded columns represent the presence of *P*-gp efflux effect.

The bidirectional transport results of the 41 drugs tested in LLC-PK1 MOCK/MDR1 cell models were summarized in Table 1. The permeability ranking of 41 compounds in the LLC-PK1-MDR1 model demonstrated a bimodal distribution, segregating the compounds into two distinct cohorts (Figure 2): 28 high-permeability drugs (P_app_ > 3 × 10^−^⁶ cm/s) and 13 low-permeability drugs (P_app_ < 3 × 10^−^⁶ cm/s). 9 compounds displayed polarized efflux (ER > 2), indicating that they are *P*-gp efflux substrates. Notably, the recoveries of amitriptyline, tetrandrine, sunitinib, and paroxetine exhibited far below 80%. Cell lysate extraction increased recoveries to >80%. This significant improvement suggested that nonspecific binding to cellular components and the apparatus was a primary mechanism of compound loss, although potential losses onto the Transwell membranes and plastic surfaces could not be ruled out without dedicated blank (acellular) studies.

**Table 1.** The bidirectional transport parameters of 41 tested drugs in LLC-PK1 MOCK/MDR1 monolayer model (sorted by P_**app(A-B, MDR1)**_**, mean ±** *s***, *n* = 3).**

	Compound	Cell line	P_app_ (×10^−6^ cm/s)		Recovery (%)		ER
			A-B	B-A	A-B	B-A	
1	Phenacetin	MOCK	35.2 ± 0.08	27.23 ± 1.32	118 ± 2.92	99.1 ± 1.86	0.77
		MDR1	36.6 ± 2.48	28.98 ± 1.19	119 ± 1.31	99.2 ± 1.34	0.79
2	Caffeine	MOCK	34.0 ± 1.22	34.10 ± 1.49	121 ± 2.48	101 ± 2.25	1.00
		MDR1	36.4 ± 1.42	33.26 ± 1.49	118 ± 5.78	94.9 ± 7.10	0.91
3	Atipamezole	MOCK	33.9 ± 1.72	30.19 ± 4.52	117 ± 7.46	102 ± 8.10	0.89
		MDR1	33.7 ± 5.21	27.55 ± 1.50	118 ± 11.1	107 ± 6.44	0.82
4	Nimodipine	MOCK	33.7 ± 2.08	46.24 ± 1.79	109 ± 7.08	91.7 ± 4.14	1.37
		MDR1	32.2 ± 1.83	44.54 ± 5.11	114 ± 5.22	94.1 ± 1.67	1.39
5	705	MOCK	32.1 ± 1.05	30.40 ± 1.91	119 ± 2.55	100 ± 4.32	0.95
		MDR1	31.5 ± 0.40	30.93 ± 2.17	111 ± 3.53	102 ± 3.55	0.98
6	Antipyrine	MOCK	31.4 ± 3.13	23.61 ± 1.43	116 ± 7.41	95.2 ± 2.32	0.73
		MDR1	30.9 ± 1.46	28.93 ± 1.75	113 ± 1.41	102 ± 10.05	0.93
7	Lamotrigine	MOCK	32.8 ± 2.04	25.31 ± 1.32	119 ± 6.58	102 ± 2.31	0.77
		MDR1	30.9 ± 1.72	29.70 ± 0.70	120 ± 5.55	99.2 ± 1.10	0.96
8	Dexmedetomidine	MOCK	30.8 ± 0.61	35.19 ± 2.56	111 ± 2.72	99.9 ± 5.22	1.14
		MDR1	30.2 ± 3.4	29.13 ± 3.62	112 ± 10.0	92.7 ± 8.47	0.96
9	Midazolam	MOCK	24.7 ± 0.41	31.55 ± 5.10	99.6 ± 1.73	97.4 ± 3.76	1.32
		MDR1	29.5 ± 1.00	27.02 ± 2.23	100 ± 3.00	98.7 ± 9.60	0.91
10	Diazepam	MOCK	34.1 ± 1.24	42.90 ± 1.42	118 ± 3.87	88.3 ± 4.96	1.26
		MDR1	28.6 ± 0.41	41.92 ± 1.30	110 ± 1.05	93.3 ± 7.98	1.47
11	Lidocaine	MOCK	27.3 ± 0.96	27.01 ± 0.42	101 ± 2.23	96.3 ± 1.19	0.99
		MDR1	27.3 ± 0.51	26.88 ± 0.43	103 ± 1.97	95.1 ± 0.82	0.98
12	Bupropion	MOCK	27.3 ± 2.88	23.78 ± 0.67	102 ± 4.17	93.8 ± 4.05	0.87
		MDR1	26.6 ± 2.58	19.52 ± 1.00	104 ± 7.38	89.1 ± 1.66	0.73
13	Tramadol	MOCK	27.3 ± 0.20	23.26 ± 1.43	113 ± 3.59	99.1 ± 3.48	0.85
		MDR1	26.3 ± 1.05	25.18 ± 1.10	109 ± 2.73	103 ± 5.78	0.96
14	Omeprazole	MOCK	34.2 ± 0.55	28.87 ± 1.39	119 ± 2.39	87.2 ± 1.79	0.84
		MDR1	25.4 ± 0.66	41.51 ± 2.92	113 ± 1.46	106 ± 0.51	1.63
15	Metoprolol	MOCK	30.1 ± 3.88	24.01 ± 1.39	114 ± 8.95	109 ± 3.06	0.8
		MDR1	24.5 ± 0.19	25.51 ± 0.59	116 ± 1.08	104 ± 2.28	1.04
16	Tolbutamide	MOCK	18.7 ± 1.12	17.23 ± 1.64	97.3 ± 2.82	105 ± 2.47	0.92
		MDR1	24.5 ± 0.06	22.83 ± 0.20	104 ± 1.21	98.9 ± 1.62	0.93
17	Tacrine	MOCK	30.9 ± 1.76	27.23 ± 2.27	114 ± 3.02	104 ± 9.23	0.88
		MDR1	23.6 ± 1.10	34.11 ± 2.16	105 ± 4.23	92.5 ± 2.49	1.45
18	Modafinil	MOCK	20.2 ± 0.98	15.62 ± 0.22	106 ± 2.03	99.3 ± 0.63	0.78
		MDR1	22.7 ± 0.45	23.93 ± 0.91	107 ± 3.40	99.08 ± 1.69	1.05
19	Diphenhydramine	MOCK	25.6 ± 2.21	22.54 ± 1.26	101 ± 7.46	96.71 ± 8.61	0.88
		MDR1	22.6 ± 1.21	20.83 ± 1.39	89.3 ± 3.00	90.0 ± 3.50	0.92
20	1111	MOCK	31.8 ± 3.31	27.31 ± 0.83	112 ± 6.99	104 ± 0.41	0.86
		MDR1	21.7 ± 0.51	23.3 ± 2.12	90.4 ± 1.69	100 ± 3.37	1.07
21	Melatonin	MOCK	22.4 ± 0.51	20.6 ± 0.92	105 ± 2.95	98.4 ± 2.97	0.92
		MDR1	20.8 ± 1.43	21.7 ± 1.08	93.1 ± 1.67	90.8 ± 4.80	1.02
22	Dextromethorphan	MOCK	35.5 ± 2.09	25.9 ± 0.91	122 ± 8.16	98.0 ± 7.37	0.73
		MDR1	18.8 ± 0.12	21.9 ± 3.18	93.6 ± 6.54	92.1 ± 7.25	1.17
23	Cannabinoid	MOCK	13.9 ± 0.93	10.7 ± 2.40	84.3 ± 6.02	96.8 ± 4.05	0.77
		MDR1	18.4 ± 2.53	13.7 ± 0.39	106 ± 5.62	95.2 ± 1.58	0.75
24	Propranolol	MOCK	22.8 ± 2.13	20.1 ± 0.71	90.9 ± 7.14	90.0 ± 2.36	0.88
		MDR1	16.8 ± 0.09	16.1 ± 1.46	80.7 ± 1.03	89.5 ± 3.33	0.96
25	Indomethacin	MOCK	16.1 ± 2.07	18.9 ± 0.31	109 ± 5.97	90.2 ± 2.95	1.18
		MDR1	14.9 ± 1.29	20.3 ± 2.74	99.7 ± 4.24	94.2 ± 4.85	1.36
26	S-071031B	MOCK	21.8 ± 1.58	25.3 ± 3.54	98.8 ± 4.05	89.9 ± 7.05	1.19
		MDR1	14.1 ± 1.97	26.6 ± 4.97	85.9 ± 1.55	92.6 ± 4.29	1.89
27	Verapamil	MOCK	24.0 ± 1.44	22.6 ± 1.08	110 ± 2.83	95.1 ± 1.89	0.94
		MDR1	9.45 ± 0.62	47.5 ± 4.72	106 ± 9.56	99.2 ± 1.78	5.03
28	Vinblastine	MOCK	4.33 ± 0.21	4.16 ± 0.32	86.5 ± 1.69	82.2 ± 9.37	0.96
		MDR1	2.84 ± 0.27	20.4 ± 1.81	103 ± 9.03	103 ± 5.71	7.20
29	Quinidine	MOCK	23.4 ± 0.37	17.2 ± 2.85	99.1 ± 15.6	98.6 ± 7.19	0.73
		MDR1	2.48 ± 1.06	42.9 ± 2.11	98.7 ± 14.8	111 ± 4.42	17.3
30	1655	MOCK	11.12 ± 2.33	8.63 ± 0.86	118 ± 7.25	99.5 ± 18.3	0.78
		MDR1	2.27 ± 0.30	36.4 ± 3.72	117 ± 16.0	108 ± 13.3	16.0
31	Salbutamol	MOCK	BLQ	0.21 ± 0.02	98.1 ± 2.21	107 ± 2.84	\
		MDR1	1.88 ± 0.35	2.66 ± 0.21	98.2 ± 0.36	96.2 ± 3.69	1.42
32	Cimetidine	MOCK	0.69 ± 0.07	0.28 ± 0.07	96.9 ± 13.3	90.4 ± 11.5	0.41
		MDR1	1.32 ± 0.31	33.9 ± 4.30	87.4 ± 9.65	94.2 ± 26.3	25.7
33	Digoxin	MOCK	1.91 ± 0.13	1.75 ± 0.07	95.0 ± 5.24	95.6 ± 3.23	0.80
		MDR1	1.15 ± 0.28	7.79 ± 0.91	109 ± 16.0	83.9 ± 0.36	6.90
34	Atenolol	MOCK	0.27 ± 0.01	0.20 ± 0.02	102 ± 3.17	88.9 ± 4.03	0.95
		MDR1	1.06 ± 0.09	1.35 ± 0.15	101 ± 3.47	107 ± 0.78	1.27
35	Doxorubicin	MOCK	BLQ	0.09 ± 0.01	112 ± 11.22	108 ± 9.06	\
		MDR1	0.95 ± 0.16	3.47 ± 0.39	109 ± 4.57	106 ± 0.51	3.65
36	Etoposide	MOCK	BLQ	0.16 ± 0.02	106 ± 0.11	103 ± 1.60	\
		MDR1	0.75 ± 0.10	3.68 ± 0.22	100 ± 2.47	99.5 ± 2.65	4.89
37	Methotrexate	MOCK	BLQ	BLQ	85.8 ± 4.58	86.7 ± 17.5	\
		MDR1	0.55 ± 0.10	0.45 ± 0.05	83.3 ± 5.67	81.4 ± 11.2	0.81
38	Amitriptyline	MOCK	9.98 ± 0.69	13.8 ± 1.58	50.0 ± 5.98	80.6 ± 8.30	1.38
		MDR1	6.84 ± 0.72	12.7 ± 1.19	43.2 ± 2.81	78.2 ± 5.74	1.85
39	Sunitinib	MOCK	9.31 ± 0.74	6.21 ± 0.20	49.5 ± 3.17	80.5 ± 5.21	0.67
		MDR1	2.23 ± 0.21	21.8 ± 1.08	64.6 ± 4.06	91.8 ± 1.11	9.77
40	Paroxetine	MOCK	7.65 ± 0.20	14.8 ± 0.57	54.1 ± 1.86	73.9 ± 2.84	1.93
		MDR1	5.15 ± 0.55	10.9 ± 0.35	45.0 ± 0.98	60.8 ± 1.32	2.11
41	Tetrandrine	MOCK	3.15 ± 0.23	4.19 ± 0.77	21.1 ± 2.22	83.1 ± 6.26	1.33
		MDR1	1.55 ± 0.29	13.0 ± 2.24	39.1 ± 6.10	89.9 ± 12.94	8.40

“BLQ”: concentration below the lower limit of detection quantification (2 nM);

“\”: unable to be calculated.

**Figure 3.**
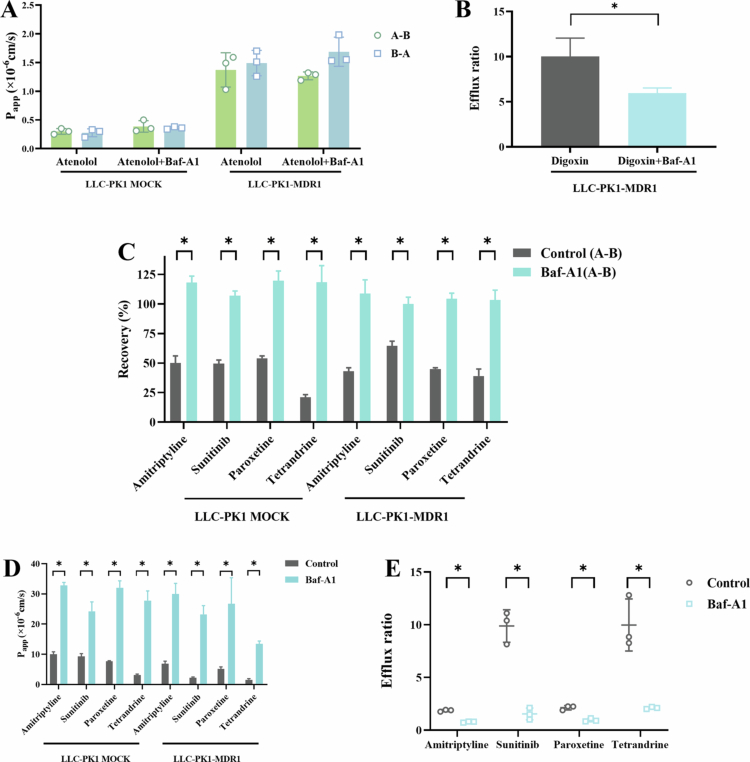
The corrective effect of Baf-A1 on the transcellular travel of intracellular accumulated drugs across LLC-PK1 MOCK/MDR1 monolayer model (A-B) Permeability and efflux rate of positive control drugs in LLC-PK1 MOCK/MDR1 cells with and without Bafilomycin A1; (C) Recovery rate of 4 intracellular accumulated drugs in LLC-PK1 MOCK/MDR1 cells with and without Bafilomycin A1; (D-E) Permeability and efflux rate of 4 intracellular accumulated drugs in LLC-PK1 MOCK/MDR1 cells with and without Bafilomycin A1.

**The role of bafilomycin A1 in LLC-PK1-MOCK/MDR1 models to block the intracellular drug accumulation.** Initial validation with reference substrates (atenolol, metoprolol, digoxin) confirmed that Baf-A1 did not compromise monolayer integrity (P_app(A-B)_ of atenolol: 0.39 × 10^−^⁶ cm/s in MOCK vs. 1.26 × 10^−^⁶ cm/s in MDR1; no significant change vs. untreated controls, Figure 3A). However, digoxin efflux in MDR1 cells decreased modestly (ER: 9.73 → 6.0, Figure 3B), suggesting partial attenuation of *P*-gp activity without structural disruption.

After adding Bafilomycin A1, by inhibiting the effect of lysosomes on the intracellular accumulation of drugs, the recoveries of sunitinib, paroxetine, tetrandrine and amitriptyline in the bidirectional transport experiments all reached the acceptable range of 80−120% (Figure 3C). Importantly, corrected permeability values revealed enhanced passive diffusion (P_app(A-B)_ and reduced efflux dependency (ER) in the MDR1 model (Figure 3D, E). These data confirm that lysosomal sequestration—not barrier dysfunction—underlies low recovery, and validate Baf-A1 as a critical tool for distinguishing true permeability from artifactual intracellular retention.

**Figure 4.**
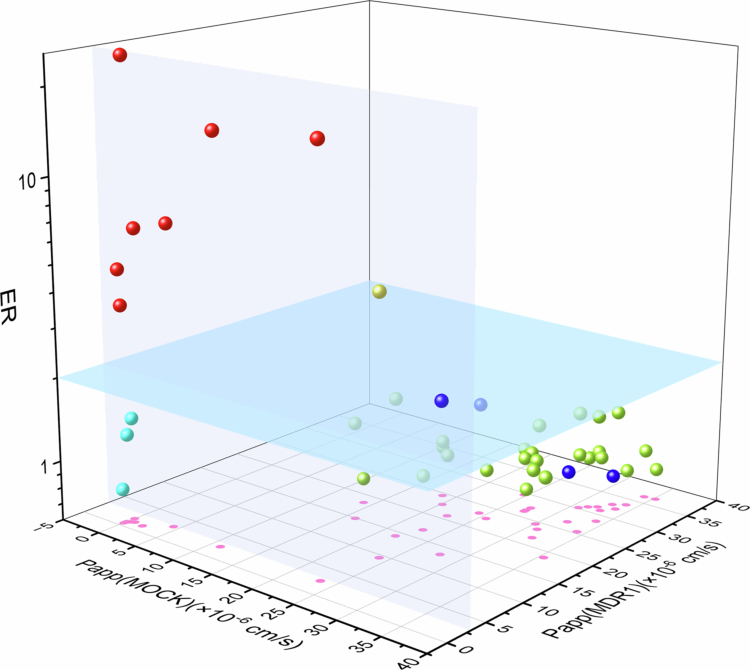
Classification of 41 tested drugs on LLC-PK1 MOCK/MDR1 model for membrane permeability and transport mechanism. The cyan spheres represent drugs with low membrane permeability that undergo passive diffusion. The green spheres represent drugs with moderate and high membrane permeability that undergo passive diffusion. The red spheres represent drugs with low membrane permeability caused by being *P*-gp substrates. The yellow spheres represent drugs that are *P*-gp substrates but have relatively good membrane permeability.

**Integration of LLC-PK1 MOCK/MDR1 model for mechanism categorization of Barrier transport.** By comparing the P_app(A-B)_ values, ER values, and recoveries in this study, 41 test compounds on the LLC-PK1 MOCK/MDR1 monolayer cell models, it was found that the 41 tested drugs covered characteristics including different permeability abilities, efflux effects, and recoveries on the monolayer model. Based on the P_app(A-B)_ values in the LLC-PK1-MDR1 cell model, 37 drugs with a recovery rate of 80% or higher were divided into two groups: the moderate-to-high permeability group (27 drugs, P_app(A-B)_ ≥ 3 × 10^−^⁶ cm/s), and the low permeability group (10 drugs, P_app(A-B)_ ≤ 3 × 10^−^⁶ cm/s). Subsequently, according to the ER values, 37 drugs were further divided into 4 quadrants in 2 dimensions to evaluate the impact of efflux transporters (Figure 4).

For the remaining 4 drugs with recoveries below 80%, the results obtained from the transport experiments with the addition of Bafilomycin A1 were incorporated into the analysis. The total 41 drugs can be classified into 4 quadrants. Among them, well permeable drugs mediated by passive diffusion account for 63.4%, and there are three passively low-permeable drugs potentially via the paracellular pathway. Drugs involving *P*-gp accounted for approximately 22%. In the involvement of *P*-gp, the permeability of different drugs varied (Figure 4).

### Brain distribution of 41 drugs in rats

**Unbound fractions of test drugs in plasma (f**_**u,plasma**_**) and brain tissue (f**_**u,brain**_**).** For compounds with existing rat plasma or brain protein-binding data from literature or internal studies, values were directly adopted. The remaining compounds (19 in brain tissues and 11 in plasma) were analyzed using rapid equilibrium dialysis.

**Free brain-to-plasma distribution coefficients (K_*p*,uu,brain_) of 41 drugs**. Brain distribution coefficients (K_*p*,uu,brain_) were calculated for 41 drugs. Data for 32 compounds were compiled from literature(Sugita et al. 1984; Le Quellec et al. 1994; Cheng et al. 2002; Fridén 2009; O’Brien et al. 2012; Wanek 2015; Bohnert 2016; Mihajlica et al. 2018; Li 2019; Li 2020; Nicolaï 2020; Bansal et al. 2023; Wang 2024) and internal datasets (previous in-house research data from our laboratory). For the remaining 9 drugs, tissue distribution experiments were conducted in rats following intravenous administration (1 mg/kg). Plasma and brain concentration-time profiles were generated, and AUC_(0-t)_ values for both matrices were calculated using WinNolin 8.1. K_*p*,brain_ was derived as the ratio of brain-to-plasma AUC_(0-t)_.

K_*p*,uu,brain_ values were computed by normalizing K_*p*,brain_ to unbound fractions (f_u,plasma_ and f_u,brain_). For drugs with > 99% protein binding, f_u_ was assigned a default value of 0.01. Final results, including all experimentally derived and literature-based values, are presented in Table 2.

**Table 2.** The protein binding and brain exposure data of tested drugs.

Compound	Protein binding in plasma (%)	Protein binding in brain tissue (%)	K_*p*, Brain_	K_*p*,uu,Brian_	Reference
705	94.5^*a*^	91.8 ± 0.51	0.53^*a*^	0.79	
1111	90.7^*a*^	97.5 ± 0.28	8.96^*a*^	2.21	
1655	57.5^*a*^	76.9 ± 0.29	0.0729^*a*^	0.0396	
Amitriptyline	−	−	20.15	0.73	(Fridén 2009)
Antipyrine	15.0 ± 5.22	19.1 ± 6.99	0.755	0.72	
Atenolol	−	−	0.07	0.026	(Fridén 2009)
Atipamezole	−	−	4.40	1.700	(Li 2019)
Bupropion	−	−	9.78	2.000	(Fridén 2009)
Caffeine	22.8 ± 2.14	28.1 ± 11.8	0.618	0.576	(Bohnert 2016)
Cannabinoid	>99	>99^*a*^	1.30^*a*^	1.20	(Bansal et al. 2023)
Cimetidine	27.8 ± 1.73	34.5 ± 3.03	0.007	0.008	
Dexmedetomidine	95.2^*a*^	86.7^*a*^	0.80^*a*^	2.21	
Dextromethorphan	65.0 ± 0.82	96.7 ± 0.33	11.53	0.825	
Diazepam	−	−	2.28	1.07	(Fridén 2009)
Digoxin	−	−	0.029	0.010	(Mihajlica et al. 2018)
Diphenhydramine	−	−	16.25	1.05	(Fridén 2009)
Doxorubicin	75^*b*^	>99	0.291	0.012	
Etoposide	97.0^*b*^	>99	0.025	0.008	(Fridén 2009)
Indometacin	−	−	0.01	0.11	(Fridén 2009)
Lamotrigine	−	−	2.02	0.880	(Fridén 2009)
Lidocaine	64.3 ± 1.50	75.0 ± 1.01	3.863^*a*^	2.70	
Melatonin	33.0	30.0^*a*^	0.5^*a*^	0.575	(Le Quellec et al. 1994)
Methotrexate	−	−	0.004	0.006	(Fridén 2009)
Metoprolol	−	−	10.375	0.64	(Fridén 2009)
Midazolam	97.0^*a*^	97.8 ± 0.15	2.44^*a*^	1.416	
Modafinil	60^*b*^	69.2 ± 4.06	0.71	0.547	
Nimodipine	95.0^*b*^	>99	2.25	0.451	(Li 2020)
Omeprazole	95.5 ± 0.68	67.9 ± 13.85	0.15	1.095	(Cheng et al. 2002)
Paroxetine	97.9 ± 0.07	>99	3.307	1.574	(O’Brien et al. 2012)
Phenacetin	58.4 ± 0.49	58.1 ± 8.76	0.763	0.768	
Propranolol	−	−	16.37	0.61	(Fridén 2009)
Quinidine	−	−	0.32	0.036	(Nicolaï 2020)
S-071031B	−	−	23.26^*a*^	1.62^*a*^	
Salbutamol	22.0 ± 1.36	68.5 ± 1.15	0.021^*a*^	0.01	
Sunitinib	98.3 ± 0.17	>99	0.965	0.563	
Tacrine	−	−	9.56	0.78	(Fridén 2009)
Tetrandrine	−	−	24.3	0.80	(Wang 2024)
Tolbutamide	−	−	0.20	0.706	(Sugita et al. 1984)
Tramadol	−	−	5.29	1.46	(Fridén 2009)
Verapamil	−	−	0.35	0.173	(Wanek 2015)
Vinblastine	94.0 ± 1.01	>99	0.528	0.088	

*“a”*: Internal data;

*“b”*: Data from https://go.drugbank.com.

The results demonstrated that variations in unbound drug distribution were critically linked to specific barrier transport mechanisms. Compound 705 exemplified passive diffusion dominance, with its K_*p*,uu,brain_ (0.79) being moderately higher than its K_*p*,brain_ (0.53) after correction for high protein binding in both plasma and brain. In contrast, dexmedetomidine served as a clear example of active influx, achieving a K_*p*,uu,brain_ of 2.21 that substantially exceeded its K_*p*,brain_ of 0.80, indicating efficient uptake transport that overcame significant plasma protein binding (95.2%).

Conversely, classical efflux was highlighted by amitriptyline, which showed high total brain distribution (K_*p*,brain_ = 20.15) but a small K_*p*,uu,brain_ (0.73). Extreme cases of this efflux included doxorubicin (K_*p*,uu,brain_ = 0.012) and etoposide (K_*p*,uu,brain_ = 0.008), where minimal unbound drug penetration persisted despite detectable total brain levels. Furthermore, cannabinoid demonstrated that near-equilibrium unbound distribution (K_*p*,uu,brain_ = 1.20) could be maintained despite >99% protein binding in both matrices. Finally, as a validation benchmark for passive diffusion without significant binding differentials or active transport, caffeine exhibited closely matched K_*p*,brain_ (0.618) and K_*p*,uu,brain_ (0.576).

### Correlation between P from LLC-PK1 MOCK/MDCK model and K of rat*p*,uu,brainapp

**Figure 5.**
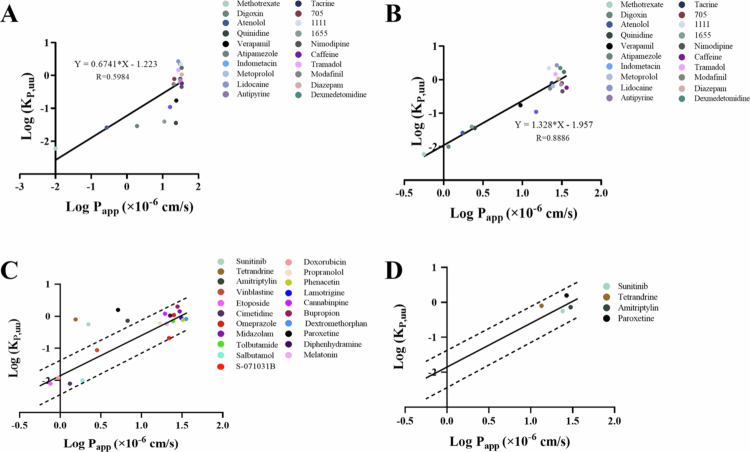
Relationship between P_app (A-B)_ and rat brain K_*p*,uu_ (A) Correlation analysis between P_app (A-B)_ values and K_*p*,uu,brian_ of tested drugs in LLC-PK1 MOCK monolayer model (*n* = 20). (B) Correlation analysis between P_app (A-B)_ values and K_*p*,uu,brian_ of tested drugs in LLC-PK1-MDR1 monolayer model (*n* = 20). (C) Validation of linear correlation accuracy for LLC-PK1-MDR1 monolayer model (*n* = 21) (Black solid line represents in vivo in vitro linear correlation predicted by the model, black dashed line represents a 2-fold error range of the model's predicted values). (D) Validation of linear correlation accuracy for LLC-PK1-MDR1 cell model with corrected Papp values of intracellular accumulation drugs (*n* = 4) (Black solid line represents in vivo in vitro linear correlation predicted by the model, black dashed line represents a 2-fold error range of the model's predicted values).

**Moderate linear correlation of P**_**app**_ **from LLC-PK1 MOCK model and K**_***p*,uu,brain**_ **of rat.** To evaluate the translational relevance of in vitro permeability, 20 drugs of 41 drugs, spanning three permeability categories, were randomly selected in a stratified manner and analyzed. A moderate linear correlation (R = 0.5984, Figure 5A) was observed between P_app(A-B)_ values from the LLC-PK1 MOCK model and in vivo K_*p*,uu,brain_ (Bansal et al. 2023). Notably, *P*-gp substrates (e.g. Compound 1655, quinidine) exhibited overestimated in vitro permeability compared to their in vivo K_*p*,uu,brain_, underscoring the LLC-PK1 MOCK model’s limited ability to account for efflux-mediated BBB restriction.

**Improved correlation using LLC-PK1 MDR1 Model**. In contrast, P_app(A-B)_ values from the LLC-PK1-MDR1 model demonstrated a robust correlation with K_*p*,uu,brain_ (R = 0.8886, Figure 5B), confirming its utility for predicting in vivo brain exposure of *P*-gp substrates. Compared with similar literature(Zhang 2006; Cucullo 2008; Li 2019; Ito 2022; Sánchez-Dengra et al. 2023), our in vitro-in vivo correlation (IVIVC) correlation is at the same level, but our method is simpler.

**Improved correlation using LLC-PK1 MDR1 Model**. The model was further validated with 21 additional drugs (Figure 5C). While 17 compounds aligned within a two-fold error range, four exhibited significant deviations. These outliers were attributed to lysosomal sequestration, a phenomenon often more pronounced in immortalized cell lines under standard culture conditions than at the specialized in vivo BBB. This led to an overestimation of its impact on permeability prediction. Reassessing these outliers using P_app(A-B)_ values after lysosomal inhibition with Baf-A1 restored agreement within the prediction range (Figure 5D). The significantly improved correlation with rat in vivo K_*p*,uu_ data-an industry-recognized surrogate for human BBB permeability-demonstrates that correcting for this in vitro-specific confounder is critical for revealing the intrinsic transport properties. This step highlights the necessity of addressing intracellular trapping to achieve accurate in vitro-in vivo extrapolation.

## Discussion

CNS disorders represent a growing global health burden, with current therapies addressing less than 5% of clinically validated targets(Nutt and Attridge 2014; GBD 2016 Neurology Collaborators 2019). Despite decades of research, CNS drug development remains hindered by two interconnected barriers: incomplete understanding of disease mechanisms and the inability to reliably predict BBB penetration during early-stage discovery(Di et al. 2013; Tsou et al. 2017; Xie et al. 2019). While innovative BBB models-including organ-on-a-chip systems and 3D bioprinted organoids- have advanced physiological relevance, their practical utility is constrained by technical complexity, low throughput, and inter-laboratory variability(Wang et al. 2017; Park 2019; Hajal 2022). These limitations render them impractical for routine drug screening in academic or industrial settings, where cost-effectiveness and scalability are paramount (Alajangi 2022).

Given the cost-effectiveness and scalability of Transwell systems (Cho 2017), we developed a 2D BBB surrogate model using LLC-PK1 MOCK and MDR1 cells, aiming to balance physiological relevance with high-throughput capability. To standardize the model, permeability of lucifer yellow (LY, ≤ 1% paracellular leakage) and TEER were concurrently monitored during culture optimization. Reported TEER thresholds vary across barrier models: 280−1000 Ω∙cm² for Caco−2 and 70−200 Ω∙cm² for MDCK monolayers(Irvine 1999; Chen 2022), but LLC-PK1 benchmarks remain undefined. In our system, LLC-PK1 MOCK and MDR1 monolayers exhibited distinct TEER peaks at day 3 (~400 Ω∙cm²) and day 6 (~100 Ω∙cm²), respectively, followed by gradual declines (Figure 1C). To ensure monolayer integrity and stability throughout the subsequent lengthy transport studies, functional experiments were intentionally performed after the peak resistance had stabilized. Therefore, the TEER values relevant to and reported alongside the permeability data are 132 ± 15 Ω·cm² (measured on day 4 for MOCK) and 89 ± 11 Ω·cm² (measured on day 7 for MDR1), reflecting the barrier condition at the time of experimentation. This reduction aligns with reports linking TEER decreases in renal epithelial cells to upregulated organic cation transporters (OCTs), which introduce transcellular ion currents without disrupting monolayer integrity (Hellinger et al. 2012). In addition, tight junction refinement-potentially via downregulation of pore-forming claudin−2-offers a compelling explanation. This would selectively reduce ionic conductance without affecting solute permeability, clarifying the TEER-LY discrepancy. Consistent LY permeability rates (P_app_ < 0.46 × 10^−^⁶ cm/s) confirmed intact tight junctions despite TEER decrease. To mitigate batch-to-batch variability, permeability assays were standardized to day 4 (MOCK) and day 7 (MDR1), corresponding to post-peak phases where LY stability (CV < 12.6%) and functional efflux were optimally balanced.

The LLC-PK1-MDR1 cell line was generated via stable MDR1 transfection, enabling systematic comparison with wild-type (MOCK) cells (Salama et al. 2006). To further validate the differences between the transfected cells (LLC-PK1-MDR1) and the wild-type cells (LLC-PK1 MOCK), we systematically compared the expression of tight junction proteins and the permeability rates of positive control drugs (high-permeability and low-permeability drugs) in both cell lines (Gupta et al. 2024). The results showed that after successful overexpression of MDR1 in LLC-PK1-MDR1 cells, the expression of tight junction proteins was lower than that in LLC-PK1 MOCK cells (Figure 1A). It is important to note that while this model forms confluent monolayers with functional tight junctions—as demonstrated herein by the presence of Claudin−7—its protein expression profile is distinct from that of the brain endothelium. Specifically, brain microvascular endothelial cells comprising the BBB are characterized by high expression of Claudin−5, a key marker largely absent in this renal model. Therefore, this system is validated for assessing *P*-gp efflux and passive diffusion but does not recapitulate the full complexity of the BBB, including endothelial-specific transporters and signaling pathways. However, the transport results of positive control drugs revealed that although the P_app(A-B)_ of atenolol in MDR1 cells reached 1.73 × 10^−^⁶ cm/s, which is higher than that in MOCK cells (0.29 × 10^−^⁶ cm/s), there was still a significant difference in permeability rates compared to metoprolol (*P* = 0.0003). To characterize the efflux features of *P*-gp, we further confirmed the efflux mechanism of digoxin by using a *P*-gp inhibitor (Figure 1H, I). The model validation results demonstrated that the LLC-PK1 MOCK/MDR1 cells established in this study, as a surrogate blood-brain barrier model, can distinguish the permeability characteristics and mechanisms of different compounds. By comprehensively integrating data from the MOCK (passive diffusion baseline) and MDR1 (efflux-active) models, we successfully established a tiered screening platform with the following key criteria: Compounds with a high P_app_ in the MOCK model and a low P_app_ in the MDR1 model are indicative of efflux substrates. When the P_app_ values are comparable in both models, it suggests that the compounds are dominated by passive diffusion. This approach enables the reliable differentiation of transport mechanisms, effectively filling a gap in conventional single-model systems.

To validate the utility of the LLC-PK1-MOCK/MDR1 model as a high-throughput surrogate for BBB permeability prediction, we systematically evaluated its performance using a diverse set of 41 drugs. This large-scale approach minimizes bias from limited sample size and enhances the robustness of in vivo correlation analysis. Bidirectional transport experiments, supported by validated LC-MS/MS quantification (calibration range: 2−2000 nM, substrate concentration: 2 µM), demonstrated that 63.4% of drugs (26/41) exhibited passive diffusion with high permeability, while 19.5% (8/41) were identified as *P*-gp substrates and 10% (4/41) showed intracellular accumulation. This broad coverage of permeability mechanisms underscores the model’s capacity to discriminate compound-specific transport behaviors. Notably, four alkaloids (sunitinib, paroxetine, amitriptyline, tetrandrine; pKa > 7) displayed abnormally low recovery (<80%), particularly in the A-B direction. Cellular lysis experiments confirmed intracellular accumulation as the primary cause, consistent with lysosomal trapping-a phenomenon where weak bases accumulate in acidic lysosomes (Bednarczyk and Sanghvi 2020). Lysosomes, a structural component of the endomembrane system, are universally presented in various tissue cells. They function as the primary cellular organelles for digestion, act as hubs for metabolic signal transmission, and participate in regulating diverse intracellular physiological and metabolic processes. Given their acidic interior, the process where drugs undergo protonation, ionization, and large-scale accumulation within lysosomes is termed "lysosomal trapping". By inhibiting lysosomal acidification with Bafilomycin A1 (Baf-A1)(Mindell 2012; Noack 2018; Dash et al. 2025), recovery rates normalized to 80−120%, accompanied by significantly improved permeability (P_app(A-B)_) and reduced ER. This correction highlights the necessity of accounting for lysosomal trapping in permeability assays, as uncorrected data would otherwise underestimate true BBB penetration potential. These findings emphasize two critical advantages of the model: 1. Integration of Baf-A1 enables differentiation between passive permeability limitations and lysosomal sequestration artifacts. 2. The good IVIVC for 37 drugs (excluding trapped compounds) validates its utility in prioritizing CNS drug candidates. For practical application, we recommend incorporating Baf-A1 during permeability assays for alkaloids or compounds with suspected lysosomal affinity, ensuring reliable prediction of brain exposure.Although lysosomal trapping plays a crucial role in the in vivo distribution of basic drugs, the physiological relevance of the model should also be considered for its in vitro evaluation. Existing literature indicates that in vitro models may either overestimate or underestimate the actual impact of lysosomal trapping, depending on whether the experimental design adequately simulates the in vivo pH gradient, cell types, and tissue barriers, etc. In the present study, a comparison between the P_app_ data of basic drugs measured in the in vitro barrier model and the in vivo BBB distribution results in rats revealed that the role of lysosomal trapping was overestimated in the in vitro model. Therefore, calibration is required, with the recovery serving as the criterion for this calibration.

Furthermore, the integration of a three-dimensional analytical framework (Figure 4)-combining passive permeability (P_app(A-B)_ in MOCK cells), efflux-modulated permeability (P_app(A-B_) in MDR1 cells), and ER-enabled precise mechanistic classification of the 41 drugs into four distinct categories: high passive permeability (e.g. metoprolol), low passive permeability (e.g. atenolol), *P*-gp-limited permeability (e.g. digoxin), and *P*-gp substrates with retained permeability (e.g. verapamil). The threshold values (P_app_ = 3 × 10^−^⁶ cm/s in MDR1 cells and ER = 2) were empirically defined to maximize mechanistic resolution. For intracellularly accumulated drugs (e.g. sunitinib), lysosomal inhibition via Baf-A1 was critical to reveal their true permeability potential after correcting for lysosomal trapping. Post-correction, these compounds predominantly clustered into category of high passive permeability, confirming that lysosomal trapping-not inherent permeability-underlies their initial misclassification in comparison to the rat in-vivo data. This triaxial strategy resolves a longstanding challenge in BBB modeling: disentangling passive diffusion from active efflux. By integration MOCK and MDR1 data, the model rapidly identifies dominant permeability mechanisms, offering an actionable roadmap for optimizing CNS drug candidates.

While in vitro Barrier surrogate models offer operational simplicity and reproducibility, their translational utility hinges on robust correlation with in vivo outcomes. To validate our model, we derived K_*p*,uu,brain_, the gold standard for quantifying free drug brain exposure from two sources: literature data (K_*p*,uu,brain_ value for 15 drugs and K_p_ vale for 16 drugs) and in-house rat PK studies (for 9 drugs lacking published data, intravenous dosing (1 mg/kg) and multi-timepoint sampling (plasma/brain) enabled K_p_ calculation). Critically, K_*p*,uu,brain_ was computed by correcting K_p_ for non-specific protein binding in plasma and brain homogenates (free fraction measured via rapid equilibrium dialysis). This step is essential under the free drug hypothesis, which posits that only unbound drug concentrations drive pharmacological activity. By integrating protein binding adjustments (11 drugs in plasma, 19 in brain homogenates), we eliminated confounding effects of differential tissue binding, isolating true BBB permeability contributions. This strategy leveraging literature evidence and controlled in vivo experiments ensured a comprehensive dataset (41 drugs) for validating the model’s predictive power.

To validate the predictive capacity of the BBB surrogate model, we first compared the in vitro permeability data (P_app(A-B)_) from LLC-PK1-MOCK and MDR1 cells with in vivo K_*p*,uu,brain_ values of 20 randomly selected drugs (representing diverse permeability profiles). While MOCK cell-derived P_app(A-B)_ exhibited moderate correlation with K_*p*,uu,brain_ (R = 0.5984), the MDR1 model that integrates barrier tightness and *P*-gp efflux achieved a robust linear correlation (R = 0.8886), demonstrating its superior physiological relevance in replicating BBB functionality.

To further confirm generalizability, the remaining 17 drugs (excluding 4 with intracellular accumulation) were used for independent validation. All validation compounds showed in vivo K_*p*,uu,brain_ values within a 2-fold error range of model predictions. Notably, for the four drugs with low recovery (<80%) due to lysosomal trapping (sunitinib, paroxetine, tetrandrine, amitriptyline), correction via lysosomal inhibition (Baf-A1) restored recovery rates and aligned their corrected P_app(A-B)_ values with in vivo outcomes (2-fold error range). This suggests that lysosomal sequestration in vitro may not fully reflect in vivo dynamics, where trapped drugs could redistribute to target tissues, leading to elevated K_*p*,uu,brain_ despite transient intracellular retention. Collectively, these results underscore two critical insights: LLC-PK1 MDR1 model which integrates *P*-gp activity and barrier properties enables accurate prediction of brain exposure, as evidenced by strong IVIVC (R = 0.8886). Low recovery (<80%) signals artifactual permeability underestimation, necessitating mechanistic correction (e.g. lysosomal inhibition) to ensure translational relevance. By addressing both passive permeability and active transport confounders, this model establishes a standardized framework for evaluating CNS drug candidates, bridging the gap between simplified in vitro assays and complex in vivo biology.

Although the developed BBB surrogate model demonstrates utility in CNS drug screening, several limitations warrant consideration. Firstly, the current framework is primarily optimized for small-molecule compounds, and its predictive capacity for large-molecule therapeutics (e.g. peptides, monoclonal antibodies) utilizing receptor-mediated transport mechanisms remains unverified. Secondly, it was speculated that endogenous expression of OCT in LLC-PK1 cells may confound permeability assessments for substrates undergoing active cellular uptake, necessitating experimental verification through OCT inhibition (e.g. verapamil co-administration) when asymmetric transport patterns (A-B > B-A) are observed. Although the model successfully addresses two critical BBB determinants including paracellular barrier integrity and *P*-gp-mediated efflux, it does not incorporate other clinically relevant transporters (BCRP, MRPs, OATP2B1) that collectively govern drug disposition at the neurovascular interface (Grube et al. 2018; Billington 2019; Shan 2022). These physiological simplifications may partially explain residual discrepancies between in vitro predictions and in vivo brain exposure profiles, particularly for compounds influenced by multiple transport mechanisms. Thirdly, this model is unable to fully recapitulate the in vivo microenvironment, this includes the absence of key cellular components such as pericytes and astrocytes, which are known to modulate BBB function. More importantly, the model cannot replicate the dynamic, disease-specific biochemical milieu. For instance, as demonstrated in Alzheimer's disease (AD) mouse models, the brain endothelium exhibits significant dysregulation, including upregulated RAGE expression which leads to increased levels of amyloid-*β* (Aβ) peptides that compete with LRP1-targeting vectors for transcytosis (Israel 2022). Such a high concentration of pathophysiological competing molecules is absent in our static in vitro system. Furthermore, the use of a porcine renal epithelial cell line inherently introduces possible species-dependent differences in transporter expression and function compared to human or rodent BBB endothelium.

## Conclusion

By leveraging the physiological hallmarks of the paracellular junction integrity and efflux transporter activity, we established a robust in vitro surrogate model using LLC-PK1-MOCK/MDR1 monolayers in a Transwell system. The model integrates rigorous optimization of experimental parameters (e.g. TEER thresholds, P_app_ validation) and mechanistic validation (e.g. lysosomal trapping correction) to enable high-throughput screening of CNS drug candidates. The in vitro-in vivo correlation observed across 60 diverse compounds offers preliminary evidence of potential utility in predicting brain exposure, particularly when accounting for artifacts related to intracellular accumulation While the model shows some untility in evaluating small-molecule permeability and *P*-gp efflux, its limitations are notable: it remains poorly suited for large biologics and compounds influenced by non-*P*-gp transporters. Additionally, the simplified cellular context used in the model lacks the structural and functional complexity of the native BBB, including interactions with pericytes, astrocytes, and the extracellular matrix, which can significantly modulate transport processes. Future efforts should focus on engineering multi-transporter cell lines (e.g. co-expressing BCRP/MRP2) and incorporating advanced technologies, such as stem cell-derived BBB organoids or machine learning-based permeability prediction, to better recapitulate human neurovascular complexity. As biomaterial science and computational modeling evolve, the integration of human physiologically relevant in vitro systems with AI-driven analytics will accelerate the rational design of brain-penetrant therapeutics, bridging critical gaps between preclinical models and clinical outcomes.

## Supplementary Material

Supplementary Material Original Image for Fig1 mock zo_1

Supplementary Material Original Image for Fig1 mock claudin_7

Supplementary Material Original Image for Fig1 MDR1 zo_1

Supplementary Material Original Image for Fig1 MDR1

Supplementary Material Original Image for Fig1 mock p_gp

Supplementary Material Original Image for Fig1 mock occludin

Supplementary Material Original Image for Fig1 MDR1 claudin_7

Supplementary Material Supplement information

Supplementary Material Original Image for Fig1 MDR1 occludin

Supplementary Material ARRIVE guidelines Author Checklist

## Data Availability

The data will be shared upon reasonable request.
