# Supplementary figures and images for: A surrogate barrier model for high-throughput blood-brain barrier permeability prediction: integrating LLC-PK1-MOCK/MDR1 Cells and lysosomal trapping correction
Source: Drug Deliv. 2025 Nov 26;32(1):2585612. doi: 10.1080/10717544.2025.2585612 (PMC12667295; doi:10.1080/10717544.2025.2585612)

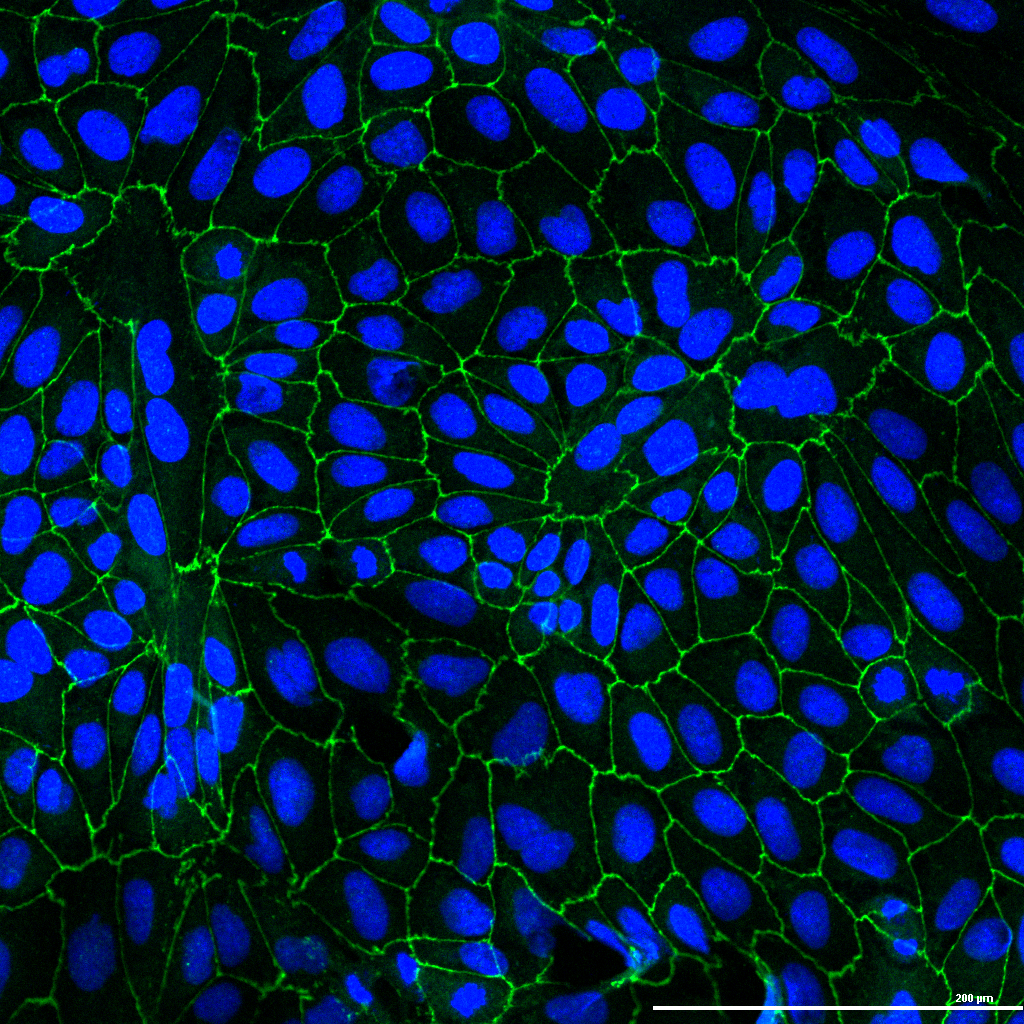

Supplement: Supplementary Material — Original Image for Fig1 mock zo_1 [file IDRD_A_2585612_SM8100.tif]

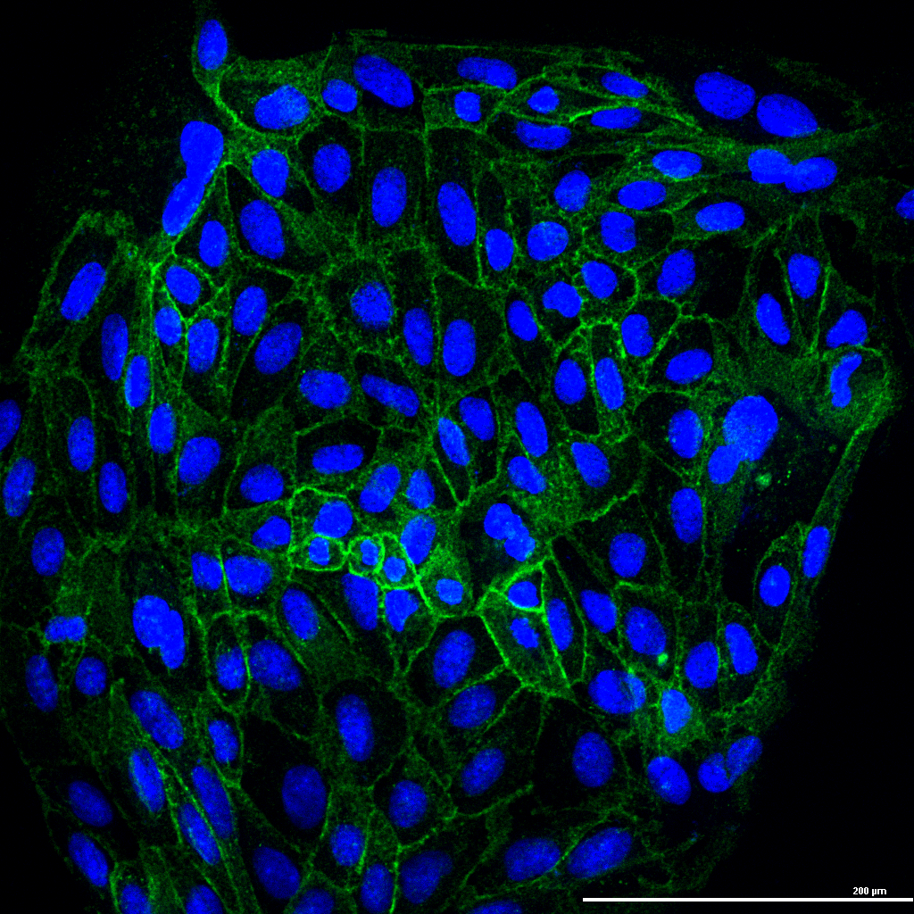

Supplement: Supplementary Material — Original Image for Fig1 mock claudin_7 [file IDRD_A_2585612_SM8099.tif]

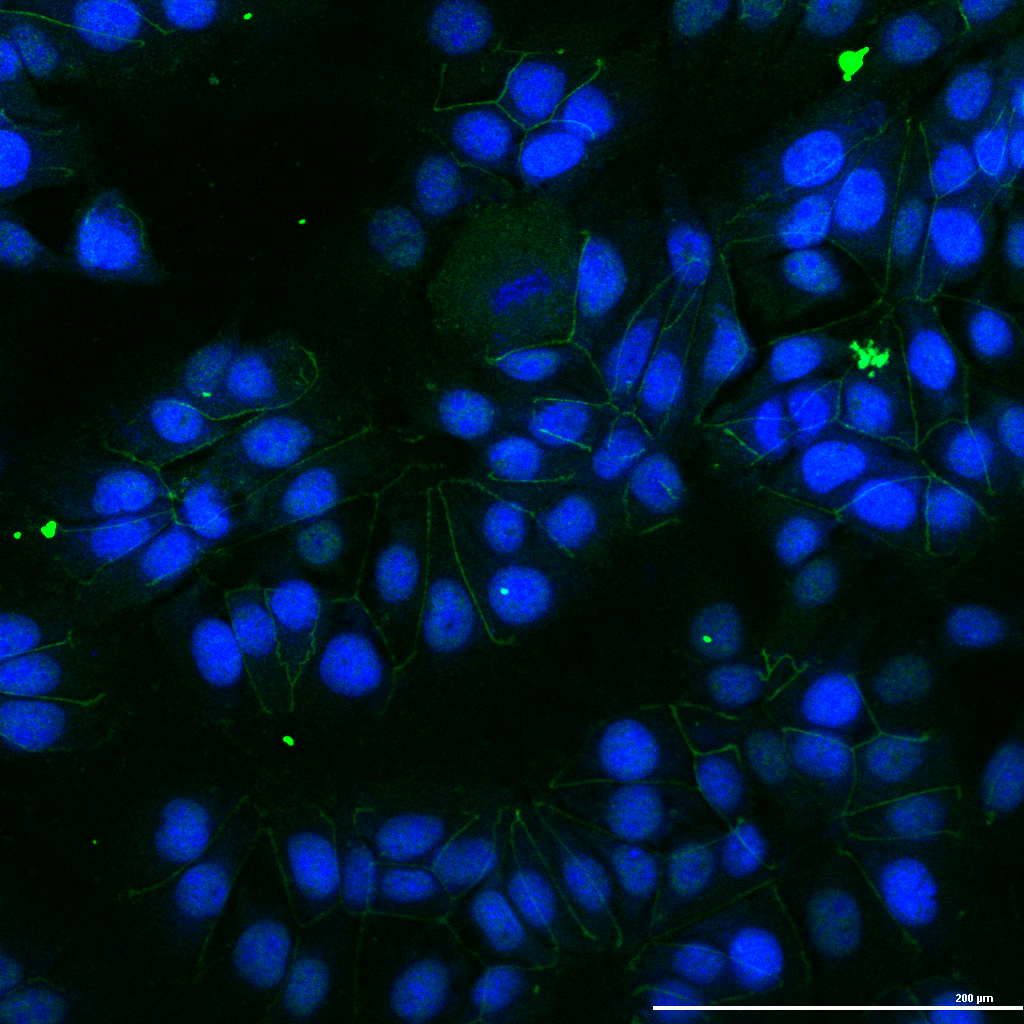

Supplement: Supplementary Material — Original Image for Fig1 MDR1 zo_1 [file IDRD_A_2585612_SM8098.tif]

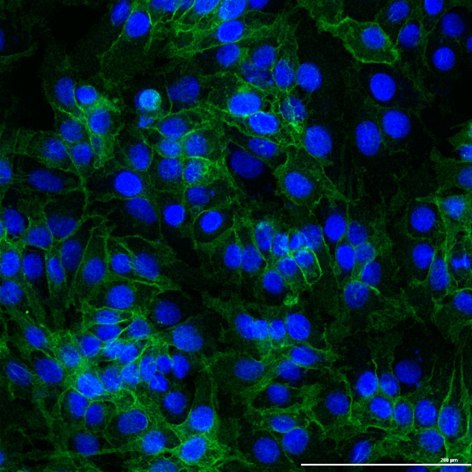

Supplement: Supplementary Material — Original Image for Fig1 MDR1 [file IDRD_A_2585612_SM8097.tif]

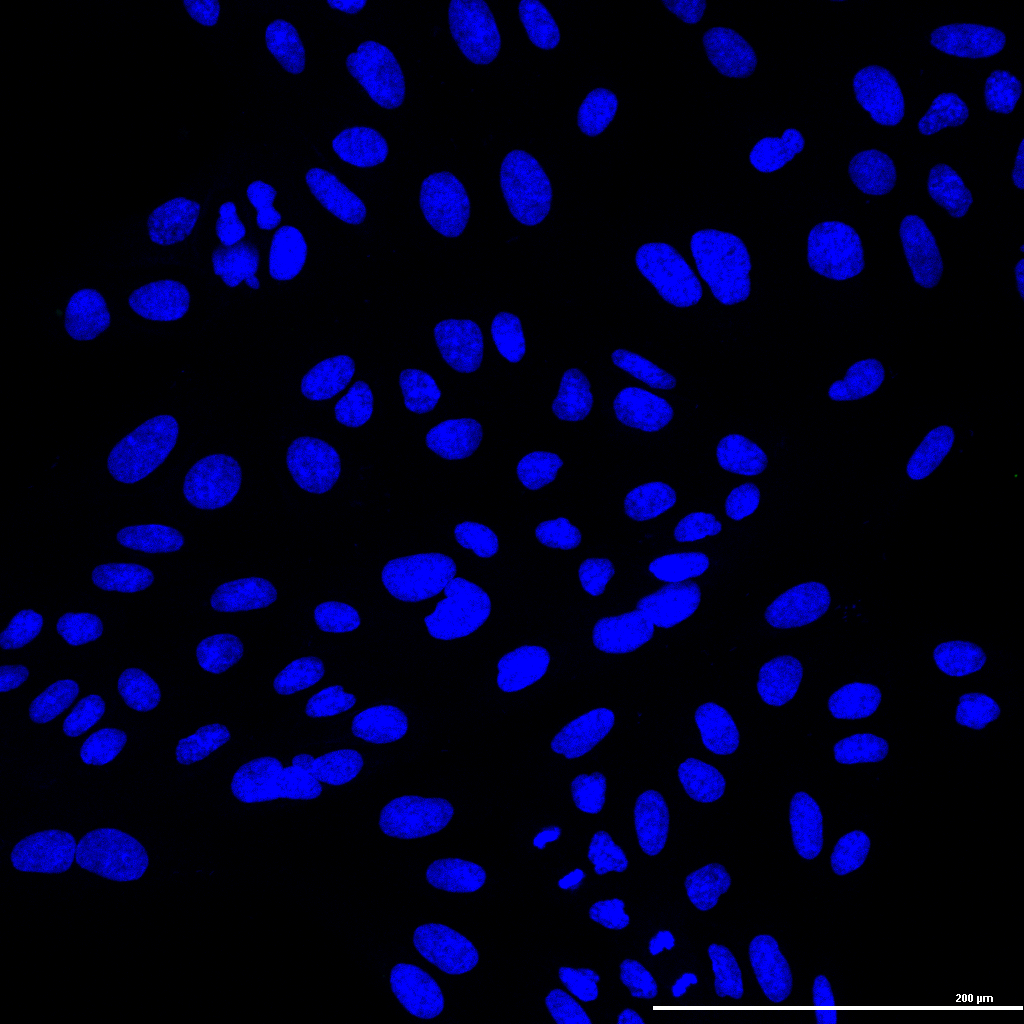

Supplement: Supplementary Material — Original Image for Fig1 mock p_gp [file IDRD_A_2585612_SM8096.tif]

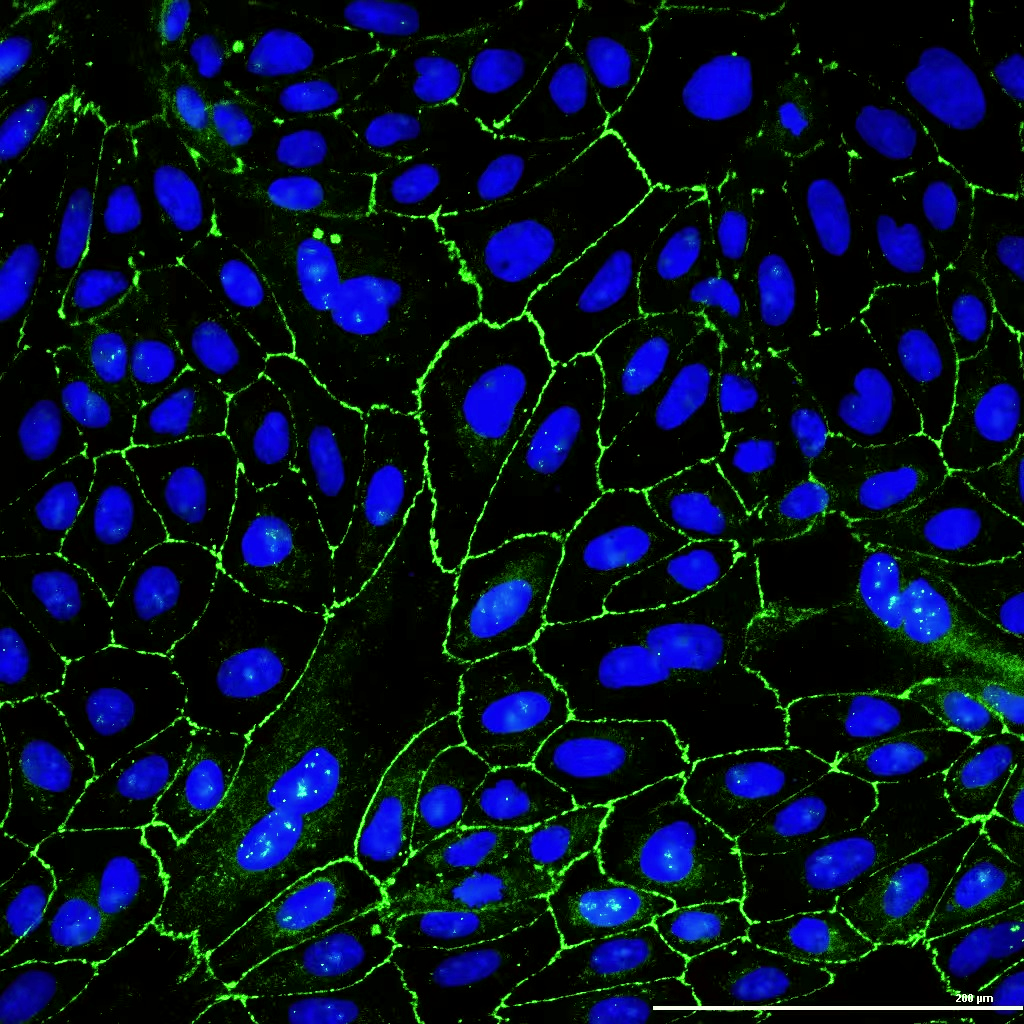

Supplement: Supplementary Material — Original Image for Fig1 mock occludin [file IDRD_A_2585612_SM8095.tif]

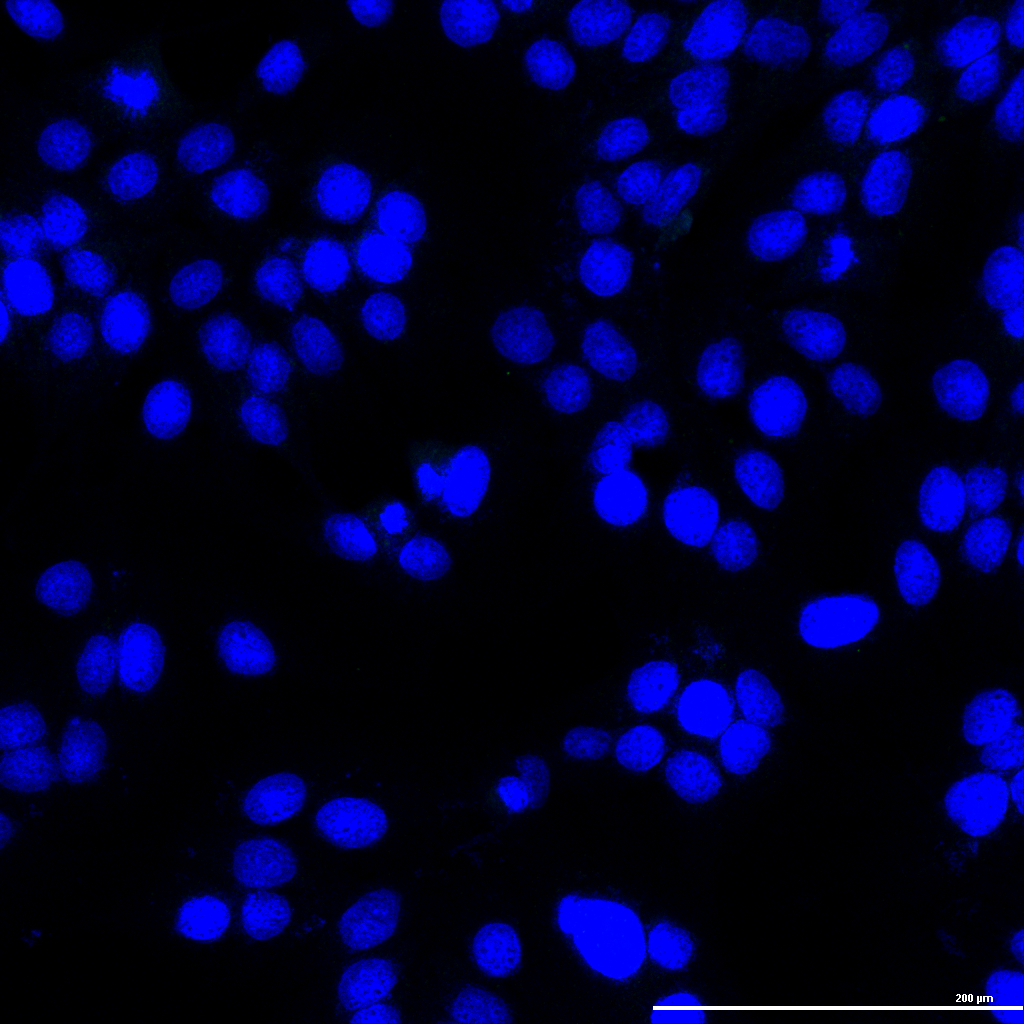

Supplement: Supplementary Material — Original Image for Fig1 MDR1 claudin_7 [file IDRD_A_2585612_SM8094.tif]

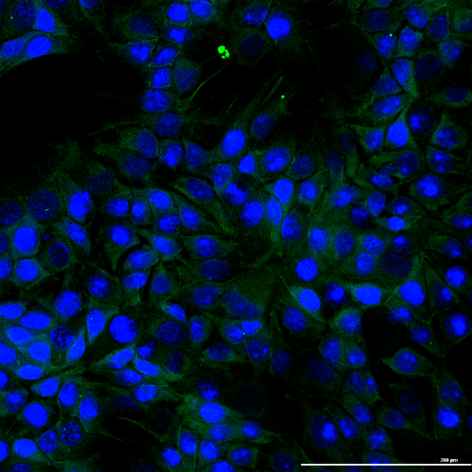

Supplement: Supplementary Material — Original Image for Fig1 MDR1 occludin [file IDRD_A_2585612_SM8092.tif]
