# Supplementary material for: A surrogate barrier model for high-throughput blood-brain barrier permeability prediction: integrating LLC-PK1-MOCK/MDR1 Cells and lysosomal trapping correction
Source: Drug Deliv. 2025 Nov 26;32(1):2585612. doi: 10.1080/10717544.2025.2585612 (PMC12667295; doi:10.1080/10717544.2025.2585612)
Supplement: Supplementary Material — Supplement information [file IDRD_A_2585612_SM8093.docx]

**A Surrogate Barrier Model for High-Throughput Brain Exposure Prediction: Integrating LLC-PK1-MOCK/MDR1 Cells and Lysosomal Trapping Correction**

Juanwen Hu#, Xue Jiang#, Cong Li, Qiannan Zhang, Xia Wu, Wenpeng Zhang, Xiaomei Zhuang*

State Key Laboratory of Toxicology and Medical Countermeasures, Beijing Institute of Pharmacology and Toxicology 100850, Beijing, China

Section 1

**Monitoring permeability of positive control drugs.**

The positive control drugs were analyzed using API5000 LCMS/MS system (AB Sciex, US). **Chromatographic conditions:** A Phenomenex C18 column (3.0 mm × 50 mm, 2.6 μm) was used; The mobile phase consisted of water (A) and acetonitrile (B), each containing 0.1 % formic acid; The elution gradient was (0.0~0.3 min, 1% B; 0.3~1.5 min, 1%~90% B; 1.5~3.5 min, 90% B; 3.5~5.0 min, 1% B). Column temperature: 40 ℃, injection volume: 5 μL. **Mass spectrum conditions:** An electrospray ionization (ESI) source operated in positive multiple-reaction-monitoring (MRM) mode was employed. Source parameters were as follows: collision-gas pressure (CAD) 8 psi, curtain-gas pressure (CUR) 35 psi, nebulizer-gas pressure (GS1) 50 psi, heater-gas pressure (GS2) 60 psi, ion-spray voltage 5500 V, and capillary temperature 550 °C. Detailed MRM transitions and optimized voltages for the positive control drugs and the internal standard (propranolol) are listed in Table. S1. **Sample treatment:** 20 µL of incubation medium were taken from either the receiver or donor compartment of the Transwell plate. After addition of 20 µL acetonitrile and 100 µL acetonitrile containing the internal standard propranolol (10 ng/mL⁻¹), the mixture was vortexed for 1 min and centrifuged at 18800 × g for 10 min. A 50 µL aliquot of the supernatant was combined with 50 µL 50 % (v/v) acetonitrile in water, vortex-mixed, and injected (5 µL) for LC–MS/MS analysis.

**Table. S1 Mass spectrometry parameters of positive control drugs**

**and the internal standard (propranolol).**

| **Compound** | **MRM** | **m/z** | | **CE**  **(eV)** | **DP**  **(eV)** | **CXP**  **(eV)** |
| --- | --- | --- | --- | --- | --- | --- |
|  |  | **Precursor Ion** | **Product Ion** |  |  |  |
| Atenolol | + | 267.0 | 145.0 | 50.0 | 76.0 | 18.0 |
| Metoprolol | + | 268.1 | 116.1 | 30.0 | 140.0 | 11.0 |
| Digoxin | + | 798.5 | 97.2 | 55.0 | 190.0 | 20.0 |
| Propranolol | + | 260.1 | 116.1 | 30.0 | 100.0 | 10.0 |

Section 2

**Bidirectional transport experiments of 41 test drugs.**

The five drug samples (1111, 705, melatonin, midazolam, and tolbutamide) were analyzed using LCMS-8060 (SHIMADZU, Japan). **Chromatographic conditions:** A Phenomenex C18 column (3.0 mm × 50 mm, 2.6 μm) maintained at 40 °C was used for all analytes. The mobile phase consisted of water (A) and acetonitrile (B), both containing 0.1% formic acid. The gradient elution program, injection volume, and total flow rate for each drug are detailed in Table. S2. **Mass spectrum conditions:** An electrospray ionization (ESI) source was operated in multiple reaction monitoring (MRM) mode. The ion spray voltage (IS) was set to 4.0 kV, the ion source temperature (TEM) to 300 °C, the collision gas (CID Gas) pressure to 270 kPa, the drying gas flow to 10 L/min, the heating gas flow to 10 L/min, and the nebulizing gas flow to 3 L/min. The mass spectrometric detection parameters for the test drugs and internal standards (chlorzoxazone and propranolol) are detailed in Table. S3. **Sample treatment:** 20 µL of incubation medium were taken from either the receiver or donor compartment of the Transwell plate. After addition of 20 µL acetonitrile and 100 µL acetonitrile containing the internal standard propranolol (10 ng/mL⁻¹), the mixture was vortexed for 1 min and centrifuged at 18800 × g for 10 min. A 50 µL aliquot of the supernatant was combined with 50 µL 50 % (v/v) acetonitrile in water, vortex-mixed, and injected (5 µL) for LC–MS/MS analysis.

**Table. S2 Liquid chromatography parameters of 5 test drugs.**

| **Compound** | **Gradient elution program** | **Injection volume (µL)** | **Total Flow (mL/min)** |
| --- | --- | --- | --- |
| 1111 | 0-0.5min B 5%，0.5-2.0min B 5%-95，2.0-2.5min B 95%，2.5-2.6min B 95%-5%，2.6-4.0min B 5% | 5 | 0.8 |
| 705 | 0-0.5min B 25%，0.5-3.3min B 25%-95%，3.3-4.0min B 95%，4.0-4.1min B 95%-25%，4.1-5.0min B 25% | 1 | 0.5 |
| Tolbutamide | 0-0.25min B 2%，0.25-1.0min B 2%-95%，1.0-1.5min B 95%，1.5-1.6min B 95%-2%，1.6-3.5min B 2% | 1 | 0.6 |
| Midazolam | 0-0.5min B10%，0.5-2.0min B 10%-90%，2.0-2.6min B 90%，2.6-2.7min B 90%-10%，2.7-4.0min B 10% | 5 | 0.8 |
| Melatonin | 0-0.5min B 5%，0.5-1.5min B 5%-95%，1.5-3.0min B 95%，3.0-3.1min B 95%-5%，3.1-4.0min B 5% | 5 | 0.5 |

**Table. S3 Mass spectrometry parameters for the five test drugs and their internal standards (chlorzoxazone and propranolol) on the LCMS-8060.**

| **Compound** | **MRM** | **m/z** | | **CE**  **(eV)** | **Q1**  **(V)** | **Q3**  **(V)** |
| --- | --- | --- | --- | --- | --- | --- |
|  |  | **Precursor Ion** | **Product Ion** |  |  |  |
| 1111 | + | 300.3 | 145.1 | -27.0 | -10.0 | -16.0 |
| 705 | + | 249.2 | 234.0 | -36.0 | -16.0 | -16.0 |
| Chlorzoxazone | ̶ | 168.1 | 132.0 | 20.0 | 20.0 | 12.0 |
| Tolbutamide | ̶ | 269.4 | 169.9 | 19.0 | 17.0 | 16.0 |
| Propranolol | + | 260.10 | 116.0 | -19.0 | -14.0 | -13.0 |
| Midazolam | + | 325.9 | 296.0 | -28.0 | -11.0 | -21.0 |
| Melatonin | + | 237.2 | 177.2 | -16.0 | -19.0 | -19.0 |

The other 36 drugs were analyzed using API5000 LCMS/MS system (AB Sciex, US). **Chromatographic conditions:** A Phenomenex C18 column (3.0 mm × 50 mm, 2.6 μm) maintained at 40 °C was used for all analytes. The mobile phase consisted of water (A) and acetonitrile (B), both containing 0.1% formic acid. The gradient elution program, injection volume, and total flow rate for each drug are detailed in Table. S4. **Mass spectrometry conditions:** Collision gas (CAD), curtain gas (CUR), nebulizing gas (GS1), ion spray voltage (IS), and capillary temperature (TEM) are detailed in Table. S5. Mass spectrometric analysis parameters for the test drugs are detailed in Table. S6. Chromatographic and mass spectrometric conditions for the positive control drugs (atenolol, digoxin, metoprolol) and propranolol are described in section 1.

**Table. S4 Liquid chromatography parameters of 36 test drugs.**

| **Compound** | **Gradient elution program** | **Injection volume (µL)** | **Total Flow (mL/min)** |
| --- | --- | --- | --- |
| Omeprazole | 0-0.3min B 5%，0.3-1.5min B 5%-95%，1.5-2.0min B 95%，2.0-2.1min B 95%-10%，2.1-3.0min B 5% | 5 | 0.8 |
| Cannabinoid | 0-0.3min B 10%，0.3-1.5min B 10%-95%，1.5-2.0min B 95%，2.0-2.1min B 95%-10%，2.1-3.0min B 10% | 5 | 0.6 |
| Verapamil | 0-0.2min B 5%，0.2-1.4min B 5%-95%，1.4-2.0min B 95%，2.0-2.2min B 95%-5%，2.2-3.0min B 5% | 1 | 0.4 |
| 1655 | 0-0.2min B 5%，0.2-1.4min B 5%-95%，1.4-2.0min B 95%，2.0-2.2min B 95%-5%，2.2-3.0min B 5% | 1 | 0.4 |
| Tetrandrine | 0-0.3min B10%，0.3-1.4min B 10%-90%，1.4-1.8min B 90%，1.8-1.9min B 90%-10%，1.9-3.0min B 10% | 5 | 0.6 |
| Phenacetin | 0-0.3min B 10%，0.3-1.5min B 10%-90%，1.5-2.0min B 90%，2.0-2.1min B 90%-10%，2.1-3.0min B 10% | 2 | 0.8 |
| Diphenhydramine | 0-0.3min B 5%，0.3-1.5min B 5%-95%，1.5-2.0min B 95%，2.0-2.01min B 95%-5%，2.1-3.0min B 5% | 3 | 0.6 |
| Quinidine | 0-0.3min B 10%，0.3-1.5min B 10%-90%，1.5-2.0min B 90%，2.0-2.1min B 90%-10%，2.1-3.0min B 10% | 2 | 0.8 |
| Tacrine | 0-0.3min B 5%，0.3-1.5min B 5%-95%，1.5-2.0min B 95%，2.0-2.01min B 95%-5%，2.1-3.0min B 5% | 3 | 0.6 |
| Dextromethorphan | 0-0.3min B 5%，0.3-1.5min B 5%-95%，1.5-2.0min B 95%，2.0-2.01min B 95%-5%，2.1-3.0min B 5% | 3 | 0.6 |
| Lidocaine | 0-0.3min B 1%，0.3-2.5min B 1%-90%，2.5-2.8min B 90%，2.8 -3.1min B 90%-1%，3.1 -3.5min B 15% | 5 | 0.6 |
| Salbutamol | 0-0.3min B 1%，0.3-2.5min B 1%-90%，2.5-2.8min B 90%，2.8 -3.1min B 90%-1%，3.1 -3.5min B 15% | 5 | 0.6 |
| Paroxetine | 0-0.5min B 15%，0.5-1.2min B 15%-95%，1.2-2.0min B 95%，2.0 -2.1min B 95%-15%，2.1 -3.0min B 15% | 2 | 0.8 |
| Modafinil | 0-0.5min B 20%，0.5-1.2min B 20%-95%，1.2-2.0min B 95%，2.0 -2.1min B 95%-20%，2.1 -3.0min B 20% | 5 | 0.6 |
| Amitriptyline | 0-0.3min B 5%，0.3-1.5min B 5%-95%，1.5-2.0min B 95%，2.0-2.01min B 95%-5%，2.1-3.0min B 5% | 3 | 0.6 |
| Nimodipine | 0-0.5min B 15%，0.5-1.2min B 15%-95%，1.2-2.0min B 95%，2.0 -2.1min B 95%-15%，2.1 -3.0min B 15% | 1 | 0.6 |
| Bupropion | 0-0.3min B 5%，0.3-1.5min B 5%-95%，1.5-2.0min B 95%，2.0-2.01min B 95%-5%，2.1-3.0min B 5% | 3 | 0.6 |
| Doxorubicin | 0-0.3min B 10%，0.3-1.0min B 10%-90%，1.0-2.0min B 90%，2.0 -2.1min B 90%-10%，2.1 -3.0min B 10% | 5 | 0.6 |
| Caffeine | 0-0.3min B 5%，0.3-1.5min B 5%-95%，1.5-2.0min B 95%，2.0-2.01min B 95%-5%，2.1-3.0min B 5% | 3 | 0.6 |
| Lamotrigine | 0-0.3min B 5%，0.3-1.5min B 5%-95%，1.5-2.0min B 95%，2.0-2.01min B 95%-5%，2.1-3.0min B 5% | 3 | 0.6 |
| Diazepam | 0-0.3min B 5%，0.3-1.5min B 5%-95%，1.5-2.0min B 95%，2.0-2.01min B 95%-5%，2.1-3.0min B 5% | 3 | 0.6 |
| Methotrexate | 0-0.3min B 5%，0.3-1.5min B 5%-95%，1.5-2.0min B 95%，2.0-2.01min B 95%-5%，2.01-3.0min B 5% | 3 | 0.6 |
| Sunitinib | 0-0.3min B 10%，0.3-1.0min B 10%-90%，1.0-2.0min B 90%，2.0 -2.1min B 90%-10%，2.1 -3.0min B 10% | 5 | 0.6 |
| Antipyrine | 0-0.3min B 5%，0.3-1.5min B 5%-95%，1.5-2.0min B 95%，2.0-2.01min B 95%-5%，2.1-3.0min B 5% | 3 | 0.6 |
| Vinblastine | 0-0.3min B 5%，0.3-1.5min B 5%-95%，1.5-2.0min B 95%，2.0-2.01min B 95%-5%，2.1-3.0min B 5% | 3 | 0.6 |
| Etoposide | 0-0.2min B 10%，0.2 -1.4min B 10%-90%，1.4-2.0min B 90%，2.0 -2.2min B 90%-10%，2.2 -3.0min B 10% | 5 | 0.6 |
| Cimetidine | 0-0.3min B 5%，0.3-1.5min B 5%-95%，1.5-2.0min B 95%，2.0-2.01min B 95%-5%，2.01-3.0min B 5% | 3 | 0.6 |
| Tramadol | 0-0.3min B 5%，0.3-1.5min B 5%-95%，1.5-2.0min B 95%，2.0-2.01min B 95%-5%，2.01-3.0min B 5% | 3 | 0.6 |
| Indomethacin | 0-0.3min B 5%，0.3-1.5min B 5%-95%，1.5-2.0min B 95%，2.0-2.01min B 95%-5%，2.01-3.0min B 5% | 3 | 0.6 |
| Atipamezole | 0-0.3min B 5%，0.3-1.5min B 5%-85%，1.5-2.0min B 85%，2.0 -2.01min B 85%-5%，2.01 -3.0min B 5% | 3 | 0.8 |
| Dexmedetomidine | 0-0.3min B 5%，0.3-1.8min B 5%-95%，1.8-2.2min B 95%，2.2-2.3 min B 95%-5%，2.3-3.0min B 5% | 2 | 0.8 |

**Table. S5 Mass spectrometry condition parameters of 5 test drugs.**

| **Compound** | **CAD**  **(psi)** | **CUR**  **(psi)** | **GSI**  **(psi)** | **GS2**  **(psi)** | **IS**  **(V)** | **TEM**  **(℃)** |
| --- | --- | --- | --- | --- | --- | --- |
| Omeprazole | 12 | 20 | 50 | 60 | 5500 | 550 |
| Cannabinoid | 8 | 20 | 50 | 60 | 5500 | 550 |
| Verapamil | 12 | 20 | 55 | 55 | 5500 | 450 |
| 1655 | 12 | 20 | 55 | 55 | 5500 | 450 |
| Tetrandrine | 8 | 35 | 50 | 60 | 5500 | 550 |
| Phenacetin | 8 | 20 | 50 | 60 | 5500 | 550 |
| Diphenhydramine | 12 | 20 | 50 | 60 | 5500 | 550 |
| Quinidine | 8 | 20 | 50 | 60 | 5500 | 550 |
| Tacrine | 12 | 20 | 50 | 60 | 5500 | 550 |
| Dextromethorphan | 12 | 20 | 50 | 60 | 5500 | 550 |
| Lidocaine | 7 | 35 | 55 | 55 | 5500 | 450 |
| Salbutamol | 7 | 35 | 55 | 55 | 5500 | 450 |
| Paroxetine | 12 | 20 | 50 | 60 | 5500 | 550 |
| Modafinil | 7 | 35 | 55 | 55 | 4500 | 450 |
| Amitriptyline | 12 | 20 | 50 | 60 | 5500 | 550 |
| Nimodipine | 7 | 35 | 55 | 55 | 4500 | 450 |
| Bupropion | 12 | 20 | 50 | 60 | 5500 | 550 |
| Doxorubicin | 8 | 35 | 55 | 55 | 4500 | 450 |
| Caffeine | 12 | 20 | 50 | 60 | 5500 | 550 |
| Lamotrigine | 12 | 20 | 50 | 60 | 5500 | 550 |
| Diazepam | 12 | 20 | 50 | 60 | 5500 | 550 |
| Methotrexate | 12 | 20 | 50 | 60 | 5500 | 550 |
| Sunitinib | 8 | 35 | 55 | 55 | 4500 | 450 |
| Antipyrine | 12 | 20 | 50 | 60 | 5500 | 550 |
| Vinblastine | 12 | 20 | 50 | 60 | 5500 | 550 |
| Etoposide | 8 | 35 | 55 | 55 | -4500 | 450 |
| Cimetidine | 12 | 20 | 50 | 60 | 5500 | 550 |
| Tramadol | 12 | 20 | 50 | 60 | 5500 | 550 |
| Indomethacin | 12 | 20 | 50 | 60 | 5500 | 550 |
| Atipamezole | 8 | 20 | 50 | 60 | 5500 | 550 |
| Dexmedetomidine | 8 | 20 | 50 | 60 | 5500 | 550 |

**Table. S6 Mass spectrometry parameters of 36 test drugs and their internal standards (Tolbutamide, Buspirone).**

| **Compound** | **MRM** | **m/z** | | **CE**  **(eV)** | **DP**  **(eV)** | **CXP**  **(eV)** |
| --- | --- | --- | --- | --- | --- | --- |
|  |  | **Precursor Ion** | **Product Ion** |  |  |  |
| Omeprazole | + | 346.3 | 198.4 | 16.0 | 66.0 | 18.0 |
| Cannabinoid | + | 315.5 | 193.3 | 30.0 | 130.0 | 11.0 |
| 1655 | + | 466.2 | 393.4 | 30.0 | 155.0 | 20.0 |
| Verapamil | + | 455.3 | 165.1 | 29.0 | 100.0 | 11.0 |
| Tetrandrine | + | 623.7 | 381.2 | 54.0 | 240.0 | 20.0 |
| Phenacetin | + | 180.2 | 110.1 | 29.0 | 71.0 | 6.0 |
| Quinidine | + | 235.2 | 307.2 | 32.0 | 70.0 | 22.0 |
| Modafinil | + | 274.2 | 167.3 | 30.0 | 100.0 | 10.0 |
| Nimodipine | + | 419.2 | 343.1 | 15.0 | 160.0 | 10.0 |
| Doxorubicin | + | 544.2 | 396.9 | 17.0 | 240.0 | 20.0 |
| Sunitinib | + | 399.0 | 283.2 | 20.0 | 230.0 | 20.0 |
| Etoposide | ̶ | 587.2 | 381.0 | -18.0 | -280.0 | -15.0 |
| Tolbutamide | ̶ | 269.4 | 169.9 | -23.0 | -42.0 | -13.0 |
| Bupropion | + | 240.4 | 184.1 | 17.0 | 113.0 | 18.0 |
| Tramadol | + | 264.3 | 58.0 | 36.0 | 97.0 | 15.0 |
| Amitriptyline | + | 278.4 | 91.2 | 41.0 | 142.0 | 20.0 |
| Lamotrigine | + | 256.3 | 211.3 | 36.0 | 210.0 | 11.0 |
| Antipyrine | + | 189.3 | 131.4 | 30.0 | 160.0 | 11.0 |
| Methotrexate | + | 455.3 | 308.2 | 30..0 | 188.0 | 34.0 |
| Vinblastine | + | 811.5 | 224.2 | 60.0 | 320.0 | 24.0 |
| Atipamezole | + | 213.1 | 117.0 | 37.0 | 246.0 | 16.0 |
| Caffeine | + | 195.1 | 138.2 | 26.0 | 159.0 | 8.0 |
| Cimetidine | + | 253.1 | 159.0 | 10.0 | 148.0 | 20.0 |
| Indomethacin | + | 358.0 | 139.0 | 7.0 | 175.0 | 16.0 |
| Diazepam | + | 258.4 | 193.2 | 7.0 | 200.0 | 10.0 |
| Dextromethorphan | + | 272.1 | 215.0 | 33.0 | 101.0 | 42.0 |
| Diphenhydramine | + | 256.2 | 167.2 | 20.0 | 85.0 | 18.0 |
| Tacrine | + | 199.2 | 171.2 | 41.0 | 200.0 | 23.0 |
| Lidocaine | + | 235.3 | 86.1 | 35.0 | 240.0 | 11.0 |
| Salbutamol | + | 240.2 | 148.3 | 24.0 | 120.0 | 11.0 |
| Paroxetine | + | 330.4 | 192.1 | 31.0 | 160.0 | 18.0 |
| Dexmedetomidine | + | 200.97 | 94.9 | 41.0 | 161.0 | 36.0 |
| Buspirone | + | 386.4 | 122.2 | 43.0 | 180.0 | 16.0 |

Section 3

**Tab. S7 Recovery rate of 4 intracellular accumulation drugs. (mean±SD, n=3)**

| **Compound** | **LLC-PK1 Cell line** | **Recovery (%)** | |
| --- | --- | --- | --- |
|  |  | **A-B** | **B-A** |
| Amitriptyline | MOCK | 50.03±5.98 | 80.58±8.30 |
|  | MDR1 | 43.15±2.81 | 78.19±5.74 |
| Sunitinib | MOCK | 49.49±3.17 | 80.45±5.21 |
|  | MDR1 | 64.55±4.06 | 91.82±1.11 |
| Paroxetine | MOCK | 54.09±1.86 | 73.89±2.84 |
|  | MDR1 | 45.02±0.98 | 60.78±1.32 |
| Tetrandrine | MOCK | 21.06±2.22 | 83.10±6.26 |
|  | MDR1 | 39.08±6.10 | 89.93±12.94 |

**Tab. S8 Recovery rate after correction of intracellular accumulation data. (mean±SD, n=3)**

| **Compound** | **LLC-PK1 Cell line** | **Recovery (%)** | |
| --- | --- | --- | --- |
|  |  | **A-B** | **B-A** |
| Amitriptyline | MOCK | 80.01±0.69 | 90.04±9.68 |
|  | MDR1 | 88.40±4.36 | 92.18±7.28 |
| Sunitinib | MOCK | 99.47±12.79 | 89.25±8.92 |
|  | MDR1 | 116.46±1.32 | 96.97±1.32 |
| Paroxetine | MOCK | 97.56±1.99 | 92.70±4.03 |
|  | MDR1 | 91.86±6.70 | 97.63±2.74 |
| Tetrandrine | MOCK | 54.11±7.24 | 93.45±6.72 |
|  | MDR1 | 74.75±4.17 | 101.69±14.53 |
